# Advances in Architectural Design, Propulsion Mechanisms, and Applications of Asymmetric Nanomotors

**DOI:** 10.3390/nano15171333

**Published:** 2025-08-29

**Authors:** Yanming Chen, Meijie Jia, Haihan Fan, Jiayi Duan, Jianye Fu

**Affiliations:** 1 State Key Laboratory of Heavy Oil Processing, China University of Petroleum, Qingdao 266555, China; 2203010408@s.upc.edu.cn (Y.C.); z24030060@s.upc.edu.cn (M.J.); z24030044@s.upc.edu.cn (H.F.); s23030049@s.upc.edu.cn (J.D.); 2 College of Chemistry and Chemical Engineering, China University of Petroleum, Qingdao 266555, China

**Keywords:** asymmetric nanomotors, propulsion mechanisms, functional applications, structure–activity relationship

## Abstract

Asymmetric nanomotors are a class of self-propelled nanoparticles that exhibit asymmetries in shape, composition, or surface properties. Their unique asymmetry, combined with nanoscale dimensions, endows them with significant potential in environmental and biomedical fields. For instance, glutathione (GSH) induced chemotactic nanomotors can respond to the overexpressed glutathione gradient in the tumor microenvironment to achieve autonomous chemotactic movement, thereby enhancing deep tumor penetration and drug delivery for efficient induction of ferroptosis in cancer cells. Moreover, self-assembled spearhead-like silica nanomotors reduce fluidic resistance owing to their streamlined architecture, enabling ultra-efficient catalytic degradation of lipid substrates via high loading of lipase. This review focuses on three core areas of asymmetric nanomotors: scalable fabrication (covering synthetic methods such as template-assisted synthesis, physical vapor deposition, and Pickering emulsion self-assembly), propulsion mechanisms (chemical/photo/biocatalytic, ultrasound propelled, and multimodal driving), and functional applications (environmental remediation, targeted biomedicine, and microelectronic repair). Representative nanomotors were reviewed through the framework of structure–activity relationship. By systematically analyzing the intrinsic correlations between structural asymmetry, energy conversion efficiency, and ultimate functional efficacy, this framework provides critical guidance for understanding and designing high-performance asymmetric nanomotors. Despite notable progress, the prevailing challenges primarily reside in the biocompatibility limitations of metallic catalysts, insufficient navigation stability within dynamic physiological environments, and the inherent trade-off between propulsion efficiency and biocompatibility. Future efforts will address these issues through interdisciplinary synthesis strategies.

## 1. Introduction

Asymmetric nanomotors, representing a pivotal research domain in nanotechnology, refer to microscale/nanoscale devices capable of autonomous motion or task execution at micro/nano dimensions [1,2,3]. By harnessing environmental energy for directional propulsion, they overcome the inherent limitations of conventional nanoparticles that rely on passive diffusion. Consequently, asymmetric nanomotors have gained significant attention from the research community in recent years [4,5]. The 2016 Nobel Prize in Chemistry awarded for pioneering work in molecular machines catalyzed theoretical and technological advances in nanoscale machinery design [6,7]. Against this backdrop, research on asymmetric nanomotors has progressively shifted from fundamental motion demonstrations toward functional applications, revealing substantial potential in environmental remediation [8,9,10,11,12,13]. Notably, Zhang et al. engineered lipase-functionalized platinum–silica (Pt-SiO_2_) asymmetric nanomotors that enhance enzyme–microplastic collision frequency via autonomous propulsion, achieving 76% degradation of PET microplastics within 48 h, thereby establishing an enzyme–motor synergistic paradigm for active remediation of aquatic microplastic pollution [14].

Recent advances in materials science and nanotechnology have catalyzed the development of systematic fabrication methods for asymmetric nanomotors [15,16,17,18,19,20], such as template-assisted synthesis, where silica microspheres serve as sacrificial templates for selective deposition of metals (e.g., Pt and Au), followed by template removal to generate asymmetric architectures [21]. Furthermore, electrochemical deposition enables site-specific functionalization by controlling electrode potentials to deposit active materials on nanoparticle surfaces [22], while physical vapor deposition utilizes vacuum sputtering to create asymmetric coatings like Au/Pt asymmetric configurations on targeted particle regions [23]. Particularly noteworthy are emerging multimodal hybrid strategies, representing the synergistic integration of physical deposition with chemical conjugation techniques to engineer complex functional nanostructures [24].

**Figure 1:**
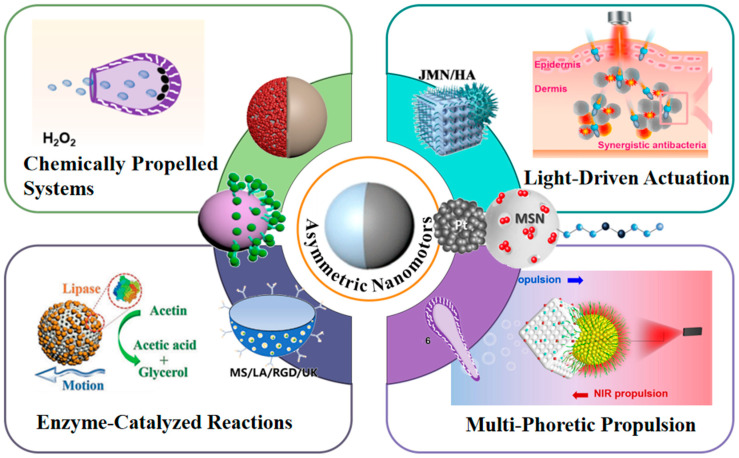
Schematic of Janus motor particles and their propulsion mechanisms.

Chemical propulsion remains the most established driving modality for nanomotors [25,26,27]. Its operation fundamentally relies on gas evolution, ion gradients, or localized concentration changes generated by chemical reactions to actuate nanoparticle motion. Classical chemical propulsion mechanisms include (1) self-diffusiophoresis, driven by autonomous chemical reactions on the nanomotor surface that generate asymmetric solute gradients, enabling directional propulsion through self-induced phoretic motion, and (2) bubble-driven propulsion, exemplified by oxygen bubbles produced from H_2_O_2_ decomposition. Illustratively, Peng Zhou’s team [28] engineered photo-crosslinked stomatocyte nanomotors wherein Pt nanoparticles catalyze H_2_O_2_ decomposition to generate O_2_ bubbles, enabling efficient locomotion even at low H_2_O_2_ concentrations. Beyond these, alternative modalities such as light-driven [29], biocatalytic [30], and hybrid propulsion systems have emerged, collectively enhancing nanomotor maneuverability and directional precision (Figure 1).

Advances in material synthesis and propulsion mechanisms have propelled [31] the functional design of nanomotors. In oncology [32], these systems notably enhance tumor penetration [33], disrupt stromal barriers, and prolong retention at lesion sites [34,35,36]. Combined with synergistic therapeutic effects and immunomodulation of the tumor microenvironment, they substantially potentiate treatment efficacy. For anti-infective applications, nanomotors demonstrate superior biofilm penetration capabilities and precise targeting performance. While current research is advancing toward clinical translation, substantial challenges remain prior to clinical deployment [37,38].

In the field of active colloids and nanomotors, the past five years have witnessed an explosive growth in the review literature. Notably, Wei Wang et al. [39] systematically reviewed the fundamental theories, propulsion mechanisms, and application scenarios of the nanomotors. The contribution of this review lies in its pioneering proposal of the “structural asymmetry–energy conversion efficiency–functional output” to highlight the structure–activity relationship (SAR) framework. First, it critically analyzes the size confinement effects of synthesis strategies, such as templating methods. Subsequently, it proposes a novel mechanistic paradigm for motion classification based on energy gradient fields, elucidating the synergistic principles governing enzyme catalysis and photothermal actuation [40]. Finally, the review demonstrates the transformative potential of these principles in diverse applications: targeted delivery [41], microplastic degradation [42], and microelectronics restoration [43]. The conclusion highlights three pivotal breakthroughs (e.g., biomimetic energy supply) and provides a roadmap for translating asymmetric intelligent nanomachines from laboratory research to clinical applications. This framework not only transcends the single-dimensional analysis limitations of traditional reviews but also establishes quantifiable cross-scale design principles.

## 2. Unique Properties of Asymmetric Nanomotors

The unique properties of asymmetric nanomotors originate from their structural anisotropy—i.e., engineered geometric/chemical asymmetry—where starkly contrasts with isotropic nanoparticles. This anisotropy breaks spatial symmetry to transduce ambient energy (chemical, optical, etc.) into net directional motion, a capability unattainable by symmetric particles dominated solely by Brownian diffusion. Crucially, such nanomotors exhibit autonomous propulsion under specific stimuli, overcoming Brownian randomization via non-equilibrium effects or non-reciprocal actuation mechanisms.

Their motion demonstrates high controllability: velocity modulation via stimulus intensity (e.g., fuel concentration or optical power), on/off switching by external triggers, and programmable navigation using fields (e.g., electric or optical). This controllability extends to environmental adaptability, where multimodal propulsion (hybrid designs responsive to multiple energy sources) enhances operational flexibility.

Functionally, asymmetric nanomotors achieve efficient mechanical output in low-Reynolds-number regimes (Re << 1), utilizing phoretic mechanisms or asymmetric field interactions to overcome viscous dominance. Furthermore, their structural design enables integrated multifunctionality, combining propulsion with sensing, cargo transport/release, or localized catalysis.

Nevertheless, inherent limitations persist: While structural anisotropy enables directional energy transduction, exposed active interfaces induce severe performance degradation in physiological environments. Precision asymmetric designs enable efficient propulsion, yet they require stringent nanoscale fabrication tolerances. This often results in significant batch-to-batch variability. Moreover, while lab-scale fabrication is mature, transitioning to industrial-scale production faces challenges in throughput, asymmetry precision control, and material versatility. Colloidal self-assembly shows potential but requires optimization for cost-effective, defect-free output. Critically, fuel dependency in chemically driven systems exacerbates biocompatibility risks due to residual toxins and undefined material clearance pathways.

Fundamentally, the symmetry-broken non-equilibrium state underpins their utility, but overcoming these barriers is essential for clinical translation and scalable deployment.

## 3. Nanomotor Structural Fabrication Strategies

Currently, multiple efficient synthetic strategies have been developed for asymmetric nanomotors, including soft-templating approaches, hard-template methods, physical vapor deposition (PVD), and self-assembly approaches. In the preparation strategies for asymmetric nanomotors, the industrial translation potential of various methods exhibits significant divergence. Hard-template synthesis, while offering moderate production throughput, suffers from critical limitations: prohibitively high manufacturing costs and unavoidable template residue contamination. These defects preclude compliance with biosafety standards for clinical applications, restricting its current utility primarily to fundamental research and environmental remediation.

In contrast, Pickering emulsion self-assembly technology demonstrates break-through industrial advantages. Its ultra-high throughput and cost effectiveness establish the foundation for scalable production. Particularly when employing fully biomaterial-based systems, this approach completely circumvents biocompatibility risks, thereby positioning it as the optimal pathway for manufacturing clinical-grade drug delivery systems. (Employing fully biomaterial-based systems allows this approach to completely circumvent biocompatibility risks. Consequently, it eliminates a major barrier for clinical translation. This positions the approach as the optimal pathway for manufacturing clinical-grade drug delivery systems.)

Physical vapor deposition (PVD) exhibits highly standardized and controllable process parameters, enabling thin-film deposition with near-atomic precision. However, its industrial adoption is hindered by prohibitively high equipment costs and inherently low deposition rates, resulting in unsatisfactory production efficiency for large-scale applications. These constraints severely restrict its integration into conventional industrial production lines, thereby limiting its current utilization primarily to advanced fields such as ultra-precision optical coatings and high-performance electronic devices.

Self-assembly enables the spontaneous construction of complex architectures through precise modulation of thermodynamic and kinetic parameters. Nevertheless, its susceptibility to environmental variations and prolonged assembly cycles considerably impede its applicability in continuous manufacturing processes. As a result, this technique is predominantly employed in cutting-edge applications including precision nanodevice fabrication and biomimetic material development.

Finally, a comprehensive comparative summary of the established synthesis methods discussed in this paper is presented in Table 1 for quick reference.

This section systematically analyzes structural asymmetry arising from distinct synthetic processes and further proposes compatible kinetic mechanisms for energy conversion alongside their potential functional implications.

### 3.1. Soft-Templating Method

Soft-template methods are widely employed due to their unique flexibility. This flexibility allows the templates to deform in response to growth-induced stresses, thereby avoiding fracture issues inherent in rigid templates during anisotropic growth. For synthesizing asymmetric nanomotors, tunable soft templates are typically used, including gas microbubbles, microemulsions, or micelles. Their dimensions and interfacial curvature are precisely controlled by modulating the hydrophilic–lipophilic balance (HLB), concentration, and temperature.

Following template assembly, precursors of functional nanomotor materials are introduced into the system, wherein hydrophilic precursors concentrate at the template-aqueous interface while hydrophobic components embed within the template’s hydrophobic core. Non-uniform nucleation and growth are subsequently induced via either (i) interfacial tension gradients driving directional precursor migration or (ii) template curvature differentials inducing anisotropic deposition rates. This asymmetric deposition is consolidated through in situ polymerization, sol–gel transition, or crystallization. Finally, the asymmetric architecture is liberated by gentle template dissolution or volatilization using mild solvents, preserving hollow or bowl-like morphologies.

**Figure 2:**
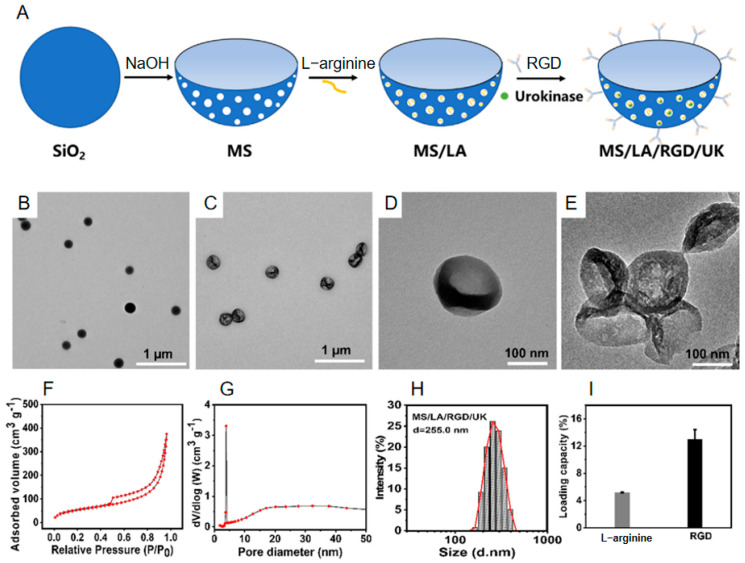
Engineering asymmetric bowl-shaped nanomotors for endogenous NO-propelled thrombolysis: synthesis evolution, structural properties, and targeted functionality. (**A**) The preparation process of MS/L-arginine (LA)/RGD peptide (RGD)/UK nanomotors. Characterizations of the nanomotors. TEM images: (**B**) spherical silica nanoparticles; (**C**,**D**) MS; (**E**) MS/LA/RGD/UK nanomotors. (**F**) N_2_ adsorption–desorption isotherm; (**G**) BJH pore size distribution curve of MS; (**H**) particle size analysis of the MS/LA/RGD/UK; (**I**) the loading capacities of LA and RGD. Experimental data are the mean ± s. d. of samples in a representative experiment (*n* = 3). Reproduced with permission from ref. [44]. Copyright 2022, Elsevier.

Yingfang Tao et al. [44] recently synthesized structurally asymmetric bowl-shaped mesoporous silica particles via a soft-template/etching coupling strategy (Figure 2A,B). This approach leverages differential hydrolysis kinetics between dual silicon sources to direct structure-selective differentiation. Under confinement by cetyltrimethylammonium bromide (CTAB) micellar templates, co-condensation of organic silicon precursor 1,2-bis (triethoxysilyl) ethane (TESPTS) and inorganic precursor tetraethyl orthosilicate (TEOS) yields core–shell hybrid spheres. Subsequent dispersion in NaOH etching solution (0.48 M) initiates selective dissolution where the inorganic silica phase is preferentially removed.

After 20 min of etching, scanning electron microscopy (SEM) reveals ≈50 nm surface indentations (Figure 2D). Termination at 30 min triggers inward contraction of the flexible organosilica framework, culminating in high-asymmetry bowl architectures with concave cavities (Figure 2E), optimized for directional propulsion. Template removal is achieved via 24 h Soxhlet extraction (90 °C) using HCl/ethanol, eliminating CTAB residues (<0.3 wt% by TGA). Final bowl-shaped mesoporous silica (MS) particles exhibit uniform diameters of 200 ± 15 nm with radial pore channels (Figure 2F,G). Subsequent amination modification via APTES functionalized the silica surface, followed by loading L-arginine (LA) as a nitric oxide (NO) precursor to enhance energy conversion efficiency. Covalent conjugation of arginine–glycine–aspartic acid (RGD) targeting peptides was achieved through carbodiimide-mediated activation, enabling specific binding to activated platelets (Figure 2I). Ultimately, urokinase (UK) was physically adsorbed within the mesopores, yielding multifunctional nanomotors that synergistically integrate thrombus-targeting (RGD), NO-propelled motility (LA), and thrombolytic therapy (UK) capabilities. Successful implementation hinges on concerted modulation of amphiphile self-assembly and silicon precursor hydrolysis dynamics. This enables the asymmetric nanomotors to harness the conversion of chemical energy into kinetic energy, propelling their targeted therapeutic function and offering a novel strategy for cardiovascular disease treatment.

### 3.2. Hard-Templating Method

In contrast to soft templating, the hard-templating approach employs rigid materials as scaffolds to transfer geometric features to target materials. This transfer is achieved through surface modification, pore filling, or deformation-induced processes. This methodology enables controlled fabrication of asymmetric nanostructures ranging from spherical asymmetric configurations to hierarchical hollow bowl-like architectures. The general pathway for preparing asymmetric nanomotors via hard templating involves three core stages: synthesis of the rigid template, asymmetric functionalization of the template surface, and selective template removal to activate propulsion. Precise control over three critical elements—template morphology design, material deposition patterns, and interfacial energy matching—is essential for constructing efficient self-propelling asymmetric structures.

**Figure 3:**
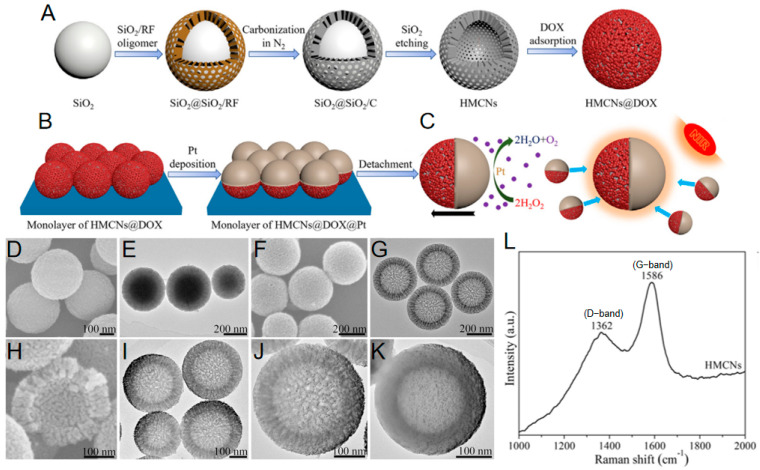
Engineering Janus HMCNs@Pt nanomotors with dual propulsion: synthesis evolution, structural hierarchy, and actuation mechanism. Schematic illustrations of (**A**) the fabrication of HMCNs and HMCNs@DOX, (**B**) the fabrication of HMCNs@DOX@Pt nanomotors, and (**C**) hybrid propulsion of the HMCNs@DOX@Pt nanomotors by the concentration gradient of the oxygen molecule from H_2_O_2_ decomposition and thermophoresis from NIR-induced temperature rise of local asymmetric NPs. SEM (**D**,**F**) and TEM (**E**,**G**) images of SiO_2_@SiO_2_/RF composites with core–shell structures (**D**,**E**) and HMCNs (**F**,**G**); (**H**) SEM image of broken HMCNs after ultrasonic treatment (power: 600 w) for more than 4 h; (**I**–**K**) TEM images of Janus HMCNs@Pt (**I**,**J**) and HMCNs@DOX@Pt (**K**) nanomotors. (**L**) Raman spectra of HMCN. Reproduced with permission from ref. [45]. Copyright 2019, Elsevier.

In a representative approach by Yi Xing et al. [45], hollow mesoporous carbon nanospheres (HMCNs) were fabricated using silica as a sacrificial template. Tetrapropyl orthosilicate served as the silicon source due to its relatively slow hydrolysis and condensation kinetics, enabling in situ formation of monodisperse silica nanoparticles with controlled dimensions that functioned as core templates. Subsequent co-deposition and co-condensation of silica oligomers with resorcinol–formaldehyde resin oligomers onto these cores yielded uniform SiO_2_@SiO_2_&RF precursor particles exhibiting core–shell structures approximately 300–400 nm in diameter (Figure 3A,B). These precursors featured solid silica cores measuring 140–240 nm surrounded by concentric striated shells approximately 80 nm thick. Carbonization under nitrogen atmosphere pyrolyzed the resin component into a carbon framework while preserving the silica phases, generating SiO_2_@SiO_2_/Carbon intermediates. Chemical etching with hydrofluoric acid then selectively dissolved all silica components, producing pure hollow mesoporous carbon nanospheres with well-defined cavities replicating the initial silica core dimensions (Figure 3F). The resulting nanostructures demonstrated uniform diameters of 320 ± 16 nm with carbon shells approximately 60 nm thick (Figure 3G), exhibiting interconnected mesopores ranging from 4 to 10 nm in diameter and a primary BJH pore size of 5.9 nm (Figure 3H). These materials possessed a high specific surface area of 1328 m^2^/g and a large pore volume of 1.01 cm^3^/g. Raman spectroscopy confirmed their carbon framework contained graphitic domains (G-band at ~1586 cm^−1^) and disordered carbon regions (D-band at ~1362 cm^−1^) (Figure 3L). The selection of tetrapropyl orthosilicate proved critical for controlling initial template dimensions and ensuring final carbon nanosphere homogeneity.

### 3.3. Self-Assembly Synthesis Approach

Traditional templating methods construct nanostructures via the spatial confinement provided by sacrificial hard templates (e.g., silica spheres and emulsion droplets) or soft templates (e.g., surfactant micelles). Their core advantage lies in precise morphological replication. However, this approach faces three fundamental inherent limitations: static structural constraints, poor interfacial compatibility, and lack of dynamic responsiveness.

To overcome these constraints, self-assembly synthesis methods have emerged. This strategy eliminates the requirement for exogenous templates, instead relying on the spontaneous synergy of intermolecular non-covalent interactions to achieve the dynamic construction of functional systems from nanoscale building blocks.

For instance, Tu et al. [46] pioneered a photo-responsive supramolecular self-assembly strategy. This method employs the amphiphilic block copolymer poly (ethylene glycol)-block-polystyrene (PEG-*b*-PS) as the self-assembly scaffold. The PEG chain terminus was covalently modified with 4-dimethylaminoazobenzene via click chemistry, yielding a photo-responsive polymer endowed with blue-light responsiveness. Azo-PEG-*b*-PS was mixed with unmodified PEG-*b*-PS at a 5:95 molar ratio and self-assembled into spherical polymer vesicles in an organic solvent.

**Figure 4:**
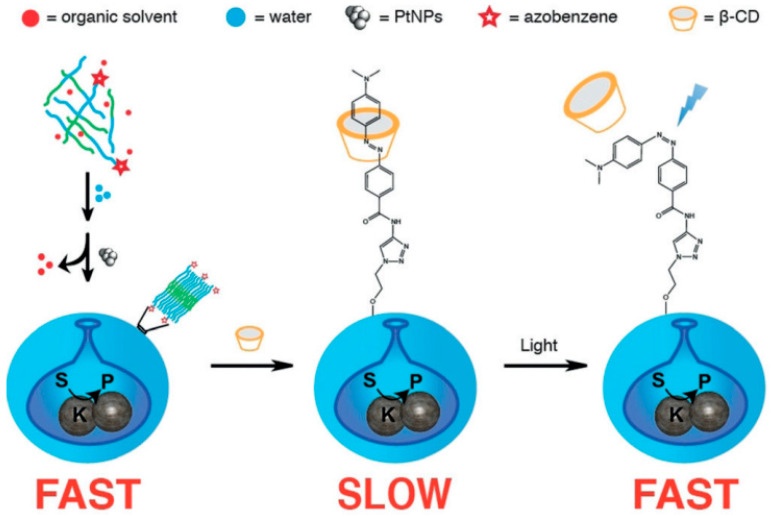
Schematic representation of a stomatocyte nanomotor with a light-responsive valve. The nanomotors are self-assembled from PEG-b-PS and functional azo-PEG-b-PS block copolymers in organic solvent, with PtNPs encapsulated within their cavities. β-CDs form host–guest complexes with trans-azobenzene groups, decreasing H_2_O_2_ diffusion due to increased steric hindrance. Blue light irradiation isomerizes trans- to cis-azobenzene, detaching β-CDs from the motor surface. This restores fuel access, increasing nanomotor speed. Reproduced with permission from ref. [46]. Copyright 2019, Wiley-VCH.

Asymmetric morphology (specifically stomatocyte structures) was subsequently induced via an osmotic shock method. First, organic solvent was removed by dialysis. Upon elevation of the internal osmotic pressure, inward buckling of the membrane occurred, resulting in the formation of bowl-shaped stomatocytes (as illustrated in Figure 4).

It is worth noting that the regulation of fuel diffusion rates through the openings of stomatocyte polymeric nanomotors via light-responsive host–guest interactions enables blue-light-triggered reversible speed modulation, which can be extended to on-demand drug release in future applications.

### 3.4. Physical Vapor Deposition

Based on its exceptional purity and density, unparalleled material versatility, nanoscale precision and conformality, superior structural control and reproducibility, and low-temperature compatibility, vacuum sputtering deposition has emerged as a highly controllable and versatile method for synthesizing asymmetric nanomotors. This physical vapor deposition (PVD) process occurs within a high-vacuum chamber, where energetic ions (commonly Ar^+^) generated in a plasma discharge bombard a target material. This bombardment ejects target atoms or clusters via momentum transfer, which subsequently traverse the vacuum environment and condense onto a strategically positioned substrate. The defining characteristic enabling asymmetry lies in precise geometric masking, oblique-angle deposition configurations, or the utilization of pre-patterned or curved substrates. These methods create non-uniform film thickness, compositional gradients, or selective material deposition across specific regions of a template particle or structure

**Figure 5:**
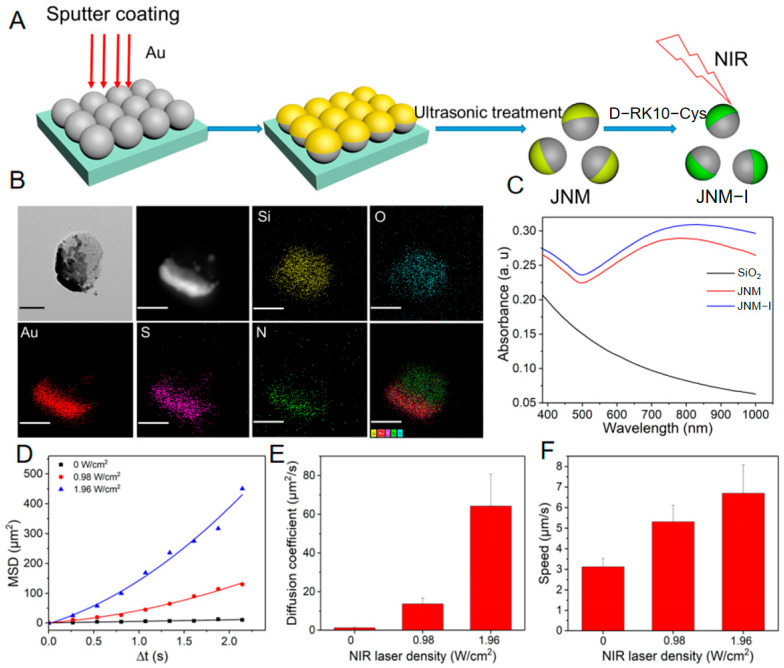
Synthesis, multimodal characterization, and kinetic behavior analysis of JNM-I nanomotor. Preparation and characterization of JNM-I. (**A**) Schematic illustration of the preparation of JNM-I. (**B**) TEM image, EDS mapping image of different elements and corresponding merged image. Scale bar: 50 nm. (**C**) Vis–NIR spectra of silica nanoparticles, JNM, and JNM-I. (**D**) Curve of MSD vs. time interval at varied laser densities. (**E**) Diffusion coefficient of JNM-I as a function of NIR laser density. (**F**) Velocity of JNM-I under different NIR laser densities. Reproduced with permission from ref. [47]. Copyright 2020, American Chemical Society.

Liu et al. [47] demonstrated the practical application of vacuum sputtering technology for fabricating structurally asymmetric nanomotors. The authors first synthesized monodisperse silica nanospheres (SiO_2_ NPs; ~106 nm) via a sol–gel method. Subsequently, utilizing a high-vacuum sputter coating system under an argon atmosphere with a constant current of 20 mA, a gold (Au) layer was directionally deposited for 100 s. This process resulted in the formation of a hemispherical Au shell (thickness: 15 ± 2 nm) on one side of the SiO_2_ NPs, establishing precise Janus asymmetry. This technique leveraged substrate confinement effects to achieve spatially selective metal deposition, yielding asymmetric nanoparticles (JNPs) with a well-defined Au-SiO_2_ interface (Figure 5A,B). Transmission electron microscopy coupled with energy-dispersive X-ray spectroscopy (TEM-EDS) mapping (Figure 5B) confirmed the hemispherical distribution of the Au element. Furthermore, diffusion coefficient analysis (Figure 5E) revealed that the JNPs fabricated by physical vapor deposition (PVD), denoted as JNP-I, achieved propulsion speeds of 5.3–6.7 μm/s under near-infrared (NIR) light irradiation, equivalent to 50–63 body lengths per second. This velocity significantly exceeded Brownian motion. The enhanced propulsion efficiency originated from the asymmetric photothermal properties conferred by the PVD process.

This asymmetric modification step not only established the asymmetric structure but also enabled efficient NIR photothermal conversion via the localized surface plasmon resonance (LSPR) effect of the Au shell (absorption peak ~800 nm). This conversion provided the driving force for “self-thermophoretic” motion (Figure 5C–F), critical for targeted delivery applications.

### 3.5. Pickering Emulsion

Solution-phase chemical synthesis demonstrates unique technical value in fabricating asymmetric nanomotors. This approach achieves spatial heterogeneity in chemical composition and function at the single-particle level through precise control over reaction kinetics, interfacial self-assembly, and region-selective deposition within liquid media.

Compared to physical vapor deposition techniques, solution-phase routes offer superior scalability prospects. Batch processes based on emulsion polymerization, flow chemistry, or continuous precipitation achieve gram-scale production per batch, reducing unit costs. Conventional physical deposition methods are fundamentally constrained by their requirement for extensive metal catalyst coverage over carrier surfaces, which severely limits available sites for stimulus-responsive functionalization. These techniques typically depend on high concentrations of toxic fuels like H_2_O_2_ to drive random Brownian motion, impeding biomedical applications [51]. Consequently, solution-phase synthesis has gained significant research interest due to its capacity for precise interfacial engineering, preservation of carrier porosity, and inherent biocompatibility [52]. Innovative approaches such as Pickering emulsion-mediated selective surface modification enable toposelective integration of functional units through interfacial assembly, providing novel pathways for constructing high-performance asymmetric motors.

**Figure 6:**
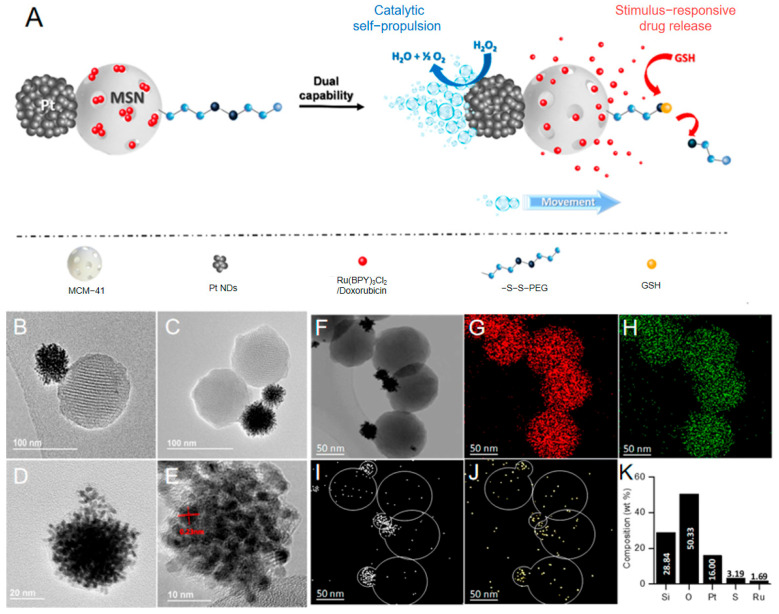
Structural hierarchy and compositional characterization of Janus Pt-MSN nanomotors. (**A**) Schematic illustration of Janus Pt-MSN nanomotors with a catalytic self-propulsion and a glutathione-mediated drug release. HR-TEM images of Janus Pt-MSNs (S_0_) (**B**,**C**) and PtNds (**D**,**E**), showing the interplanar spacing of 2.3 Å in the (111) plane of PtNd crystals. STEM image (**F**), STEM-EDX elemental mapping of Si atoms (**G**), O atoms (**H**), Pt atoms (**I**), and S atoms (**J**), and wt% composition detected (**K**) in S_1_ Janus Pt-MSNPs. Reproduced with permission from ref. [48]. Copyright 2021, American Chemical Society.

Within this context, Díez and colleagues [48] developed an advanced solution-phase synthesis strategy employing mesoporous silica nanoparticles (MSNs) as drug carriers and platinum nanodendrites (Pt NDs) as propulsion units. Their Pickering emulsion-confined reaction achieves asymmetric assembly through a sequential process (Figure 6A): MSNs are partially embedded at the paraffin/water–ethanol emulsion interface; the exposed surface is modified with (3-mercaptopropyl) trimethoxysilane; and Pt NDs are subsequently anchored via thiol bonds to form an asymmetric structure (designated S_0_). This strategy surpasses physical deposition by preserving the high surface roughness and catalytic sites of in situ-grown Pt NDs, yielding an approximately threefold increase in specific surface area (Figure 6D,E). Simultaneously, approximately 70% of the MSN surface remains available for drug loading and functionalization with stimuli-responsive gating systems like S-S-PEG linkages (Figure 6F–J). This dual-face heterostructure design enables nanomotors to achieve ultrahigh propulsion speeds of 19.4 μm/s in 0.35% H_2_O_2_, demonstrating high-efficiency energy conversion (equivalent to 426 body lengths per second per percent fuel concentration), while concurrently enabling intracellular glutathione-triggered drug release. These results underscore the distinctive advantage of solution-phase chemical synthesis in harmonizing propulsion efficiency with advanced bio-functionalization capabilities.

The ultra-rapid autonomous propulsion and directional mobility enable active negotiation of biological barriers (e.g., vascular walls and interstitial spaces), enhancing accumulation in target tissues (e.g., tumors) and promoting cellular uptake efficiency. Concurrently, the catalytic decomposition of H_2_O_2_ intrinsically modulates the tumor microenvironment through localized H_2_O_2_ depletion, conferring supplementary therapeutic benefits via antioxidant effects.

### 3.6. Single-Step Thermodynamic-Controlled Coating Method

While physical methods such as physical vapor deposition enable nanoscale-precision metal coating and anisotropic control, they rely on high-vacuum equipment and complex processes. Furthermore, the resulting rigid templates often lead to a significant reduction in specific surface area, limiting the space available for subsequent biofunctionalization modifications. In contrast, liquid-phase chemical synthesis methods offer operational flexibility and lower costs but are constrained by challenges such as difficult control over particle size uniformity, stringent reaction conditions, and randomness in surface chemical modifications. Both strategies struggle to simultaneously achieve low-cost, high-precision, and scalable fabrication, severely hindering the clinical translation of nanomotors.

In recent years, to overcome these limitations, physiochemical synthesis has emerged as a novel paradigm. This approach integrates the inherent molecular-level controllability of chemical processes with the directional regulation of physical parameters, such as free energy and contact angle. Consequently, it eliminates the need for expensive equipment typically associated with techniques like physical sputtering or microfluidics. Moreover, this method enables precise nanomotor fabrication by controllably modulating thermodynamic parameters and interfacial behavior. Such precision is clearly evidenced by the uniform products observed in TEM and SEM characterization.

**Figure 7:**
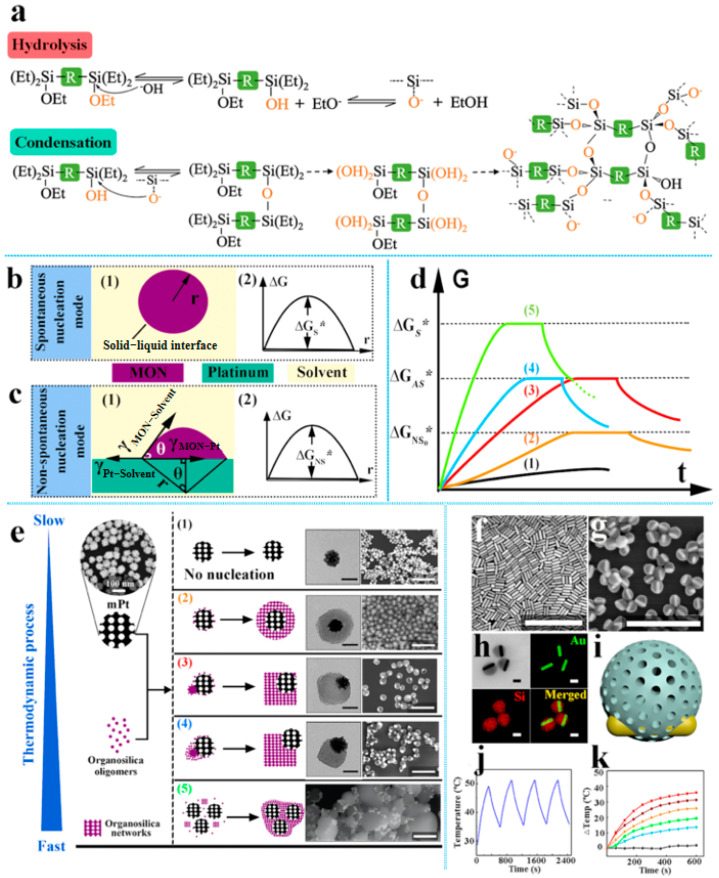
Overall process of thermodynamic-controlled coating method and its universality proof. (**a**) Hydrolysis and condensation processes of organosilanes for preparing JMNs. (**b**) Spontaneous nucleation mode (1) and matched total free energy change (2). (**c**) Non-spontaneous nucleation mode (1) and matched total free energy change (2). (**d**) Thermodynamic growth process of organosilica alkoxides for preparing polymerized MON nanostructures. (1) to (5) illustrate the five growth modes of organosilica oligomers at different ammonia concentrations. (**e**) Configuration regulation of Janus mesoporous nanomotors based on mesoporous platinum nanoparticles via process-controlled method. (**f**–**k**) Configuration and photothermal transformation effect of conceptual JMNs (CJMNs) based on gold nanorods. (**j**) Thermal cycle curve of CJMN. (**k**) Heating-rate lines graph of CJMNs, in which curves from the bottom to top match different experiments under varied materials concentrations of CJMN. Reproduced with permission from ref. [49]. Copyright 2021, American Chemical Society.

Within this framework, Chen et al. [49] developed an innovative physicochemical synthesis strategy governed by thermodynamic control. The methodology centers on precise modulation of the system’s physicochemical state through ammonia concentration (Figure 7a). Increasing ammonia concentration accelerates hydrolysis and condensation kinetics of the organosilane precursor (BTSE), thereby elevating the concentration of organosilica oligomers. This consequently increases the system’s total free energy (G)–a critical physical parameter dictating nucleation pathway evolution.

At lower G values, organosilica preferentially undergoes non-spontaneous nucleation on platinum seed surfaces (Figure 7c), forming uniformly coated core–shell structures. As G increases, the system shifts toward spontaneous nucleation (Figure 7d), triggering detachment of organosilica from the template surface. This study reveals, for the first time, the pivotal bridging role of the contact angle (θ) between thermodynamics and interfacial phenomena: θ increases with rising G, causing gradual displacement of nucleation cores away from the Pt seed interface. This shift transforms molecular arrangement from affinity-driven spreading to island-like aggregation. The synergistic G-θ mechanism drives continuous morphological evolution from exposed Pt seeds through concentric coatings and non-concentric hybrid structures to characteristic asymmetric architectures (Figure 7e).

This physicochemical regulation framework demonstrates cross-material versatility. Using the same thermodynamic control logic, the authors achieved directional conversion of gold nanorod templates into asymmetric structures. This success not only confirms the broad applicability of their approach across diverse heterogeneous interfaces (Figure 7f–k) but also demonstrates that it is a more scalable alternative compared to the methods previously described. This work not only establishes a novel single-step synthesis paradigm for nanomotors but also advances the theoretical foundation for controlled assembly of complex nanostructures by establishing quantitative correlations between reaction system thermodynamics and interfacial physical properties.

### 3.7. Multi-Step Bioconjugation

Overcoming the inherent limitations of traditional homogeneous modification strategies in reconciling autonomous propulsion with bio-specificity demands, multi-step bioconjugation synthesis leverages orthogonal chemical reactions to sequentially integrate multifunctional modules. This approach enhances structural precision and bioactive moiety retention. The strategy proves particularly critical for fabricating enzyme-driven asymmetric nanomotors, where asymmetric enzyme anchoring constitutes the fundamental basis for generating chemotactic propulsion gradients, while directional coupling of therapeutic molecules directly governs targeting efficiency. As established by Ma., Janus-mediated enzyme modification effectively mitigates Brownian motion interference [53]. Concurrently, Hu and colleagues pioneered a biomimetic platelet membrane-coating technology that established a novel paradigm for thrombus targeting [54].

**Figure 8:**
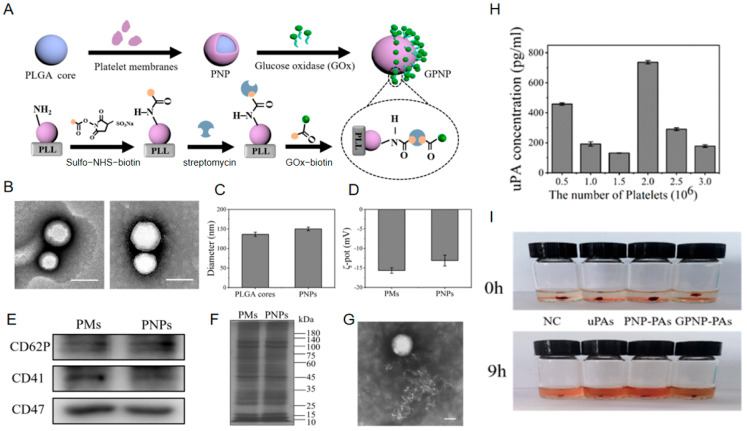
GPNP-PAs for synergistic thrombolysis: fabrication scheme, physicochemical characterization, and therapeutic efficacy validation. (**A**) Schematic diagram of the fabrication of GPNP-PAs. Characterization of nanomotors. (**B**) TEM images of unmodified PLGA cores (left) and PNPs (right). Scale bar, 100 nm. (**C**) Diameter size comparison among unmodified PLGA cores and PNPs (*n* = 3). (**D**) ζ-potential comparison among PMs and PNPs (*n* = 3). (**E**) Western blotting analysis of major proteins. (**F**) Silver stain of proteins retained on PMs and PNPs. (**G**) TEM images of GPNPs negatively stained with uranyl acetate. Scale bar, 100 nm. (**H**) Drug loading of uPAs. (**I**) Figures of in vitro thrombolytic effects of nanomotors. Reproduced with permission from ref. [50]. Copyright 2023, American Chemical Society.

Advancing this synthetic framework, Fang et al. [50] developed a GOx-propelled asymmetric platelet nanomotor (designated GPNP-PAs). Its structurally asymmetric architecture was achieved through a rigorously sequenced three-step bioconjugation protocol: First, surface modification with poly-L-lysine (PLL) induced the directional adsorption of platelet membrane-coated PLGA (Figure 8B,C) nanoparticles (PNPs) (Figure 8G). Subsequently, a biotin–streptavidin cascade reaction specifically immobilized glucose oxidase (GOx) onto one hemisphere of the particles to establish efficient energy conversion, forming the propulsion unit (GPNPs). Ultimately, thiol activation followed by Sulfo-SMCC crosslinking covalently anchored the thrombolytic drug urokinase-type plasminogen activator (uPA) onto the particle surface (Figure 8H). The methodology strictly adhered to a sequential logic of carrier functionalization, asymmetric enzyme modification, and drug conjugation. Intermediate purification steps were incorporated to ensure bioactivity preservation at each stage. This precise spatial control endowed GPNP-PAs with synergistic functions: simultaneous autonomous propulsion capability and thrombus-targeting specificity (Figure 8I), thereby providing an innovative solution for dynamic thrombolytic therapy.

## 4. Locomotion Modes

The propulsion mechanism of asymmetric nanomotors fundamentally relies on precisely engineered structural asymmetry to convert ambient energy (optical, chemical, electrical, etc.) into directional mechanical motion. This process centers on breaking spatial or temporal symmetry in microscopic systems, enabling directed force generation under thermodynamic non-equilibrium conditions. Governed by the Fluctuation Theorem and Smoluchowski Equation, Brownian motion dominates particle dynamics at micro/nanoscale dimensions. Structural asymmetry rectifies stochastic thermal motion into net directional displacement through spatially anisotropic response, an embodiment of nanoscale energy-to-motion conversion. Critically, the form of energy input dictates distinct propulsion modalities: (1) light-driven motion: enabled by photothermal/photochemical asymmetry; (2) enzyme-driven motion: dependent on asymmetric distribution of catalytic sites.

This subsection will begin by analyzing structural asymmetry and systematically categorizing the propulsion mechanisms. It will then map the underlying principles onto compatible application domains. Performance comparisons are quantitatively summarized in Table 2 for rapid reference.

### 4.1. Chemically Propelled Systems

Chemical reaction-driven propulsion stands as a key mechanism for endowing nanoparticles with autonomous motility. Its core principle lies in harnessing localized chemical reactions occurring on or within the particle to directly convert chemical energy into directed mechanical motion. Self-diffusiophoresis represents a significant physical phenomenon within this class of mechanisms: Colloidal particles achieve self-propulsion by actively generating a non-uniform chemical gradient field around themselves via surface catalytic reactions. This process operates without external fields, with the driving force originating directly from the reaction-induced asymmetric chemical potential distribution near the particle surface. Specifically, surface chemical reactions consume or produce solutes within specific regions of the particle, establishing a solute concentration gradient. This gradient induces an asymmetric spatial distribution of chemical potential at the interface, which, in turn, drives the flow of solvent molecules from regions of low chemical potential to regions of high chemical potential primarily through intermolecular interactions. This solvent flow, conversely, propels the particle in the direction opposite to the concentration gradient. Thus, chemical reactions constitute the starting point of energy conversion, while self-diffusiophoresis serves as an effective physical pathway for the direct transformation of chemical energy into mechanical energy.

**Figure 9:**
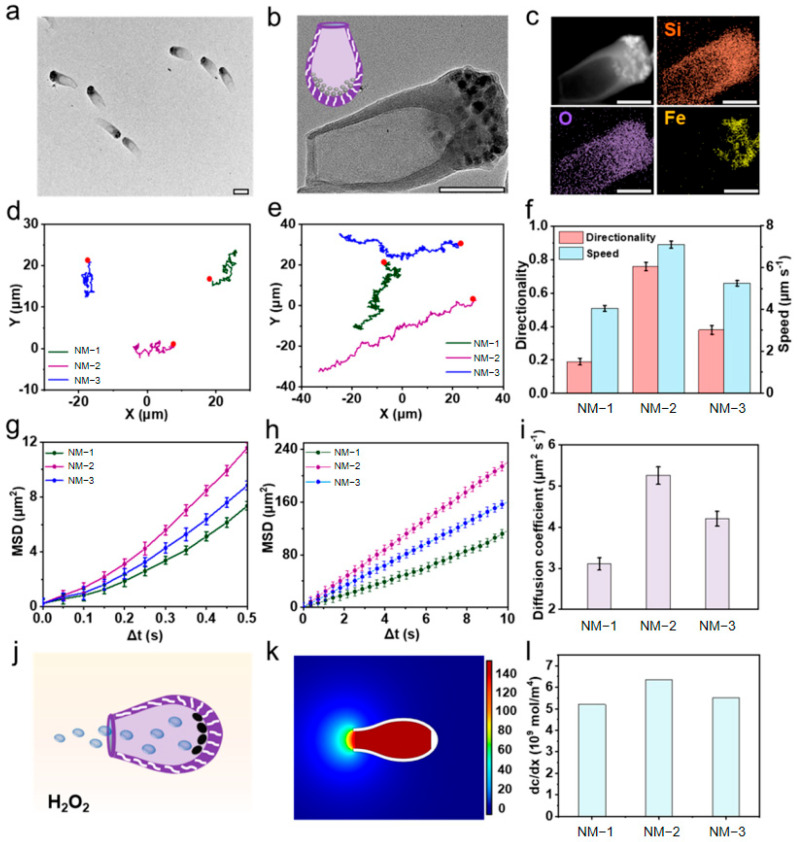
Motional behaviors of the streamlined nanomotors with different structural parameters. (**a**,**b**) TEM images with different magnifications and (**c**) element mappings of the streamlined mesoporous silica nanomotors. The inset in panel b is a 3D structural model of the Fe_3_O_4_-catalyst-loaded nanomotors. (**d**) Trajectory tracking of the nanomotors in the absence of H_2_O_2_ solution, (**e**) trajectory tracking, and (**f**) corresponding ratio of the travel distance to linear displacement and average speed of the nanomotors in 3% H_2_O_2_ solution in 10 s. (**g**,**h**) Curves of the mean square displacement versus time interval analyzed from tracking trajectories. (**i**) Effective diffusion coefficient of nanomotors measured from the slopes of the linear fitting plots of MSD curves. (**j**) Scheme illustration of the self-propelled nanomotor. (**k**) The simulated steady-state concentration gradient distribution at the opening of the streamlined hollow nanoparticles and (**l**) the steady-state concentration gradient at the opening of different nanomotors. Scale bars represent 200 nm in panel a and 100 nm in panels (**b**,**c**). Reproduced with permission from ref. [55]. Copyright 2021, American Chemical Society.

This self-diffusiophoretic mechanism is widely applied in artificial nanomotor design. A representative example is the streamlined, tadpole-shaped mesoporous silica nanomotor developed by Ma et al. [55]. In this system, Fe_3_O_4_ catalysts were selectively confined within the head cavity of tadpole-shaped nanostructures (Figure 9a–c). During catalytic decomposition of H_2_O_2_ to O_2_ and H_2_O, localized oxygen enrichment at the head region established an asymmetric concentration gradient (Figure 9j). Consequently, solvent molecules migrated from the low-concentration zone (tail) toward the high-concentration zone (head) (Figure 9k), driving the particle opposite to the concentration gradient direction (i.e., head-first propulsion).

The combination of its expansive head cavity and streamlined architecture enables efficient drug payload encapsulation and enhanced navigation through biofluids (e.g., blood), delivering substantial value for integrated theranostic applications.

### 4.2. Light-Driven Actuation

Compared to chemically propelled nanomotors, light-driven systems exhibit unique advantages in practical applications. Owing to the spatiotemporal controllability and non-contact nature of light, they enable real-time start/stop operation and directional steering of motion. Moreover, such systems operate without the need for continuous supply of chemical fuels (e.g., hydrogen peroxide), thereby avoiding potential biological interference caused by fuel toxicity, local pH fluctuations, and product accumulation. These features make light-driven nanomotors particularly suitable for long-term and high-precision biomedical operations. This energy initiates localized non-equilibrium physical fields through light–matter interactions, leveraging geometric/chemical asymmetry in Janus structures to convert photons into directional mechanical motion.

Among light-driven nanomotor systems, self-thermophoresis represents a typical propulsion mechanism. The core process of this mechanism involves the formation of an asymmetric heating field on the surface of micro/nanoparticles due to energy absorption by photothermal materials, leading to a temperature gradient (∇T) at the solid–liquid interface. This gradient induces directional fluid flow (thermo-osmotic flow), propelling particle motion. The propulsion velocity follows:

u_T_ = −D_T_∇T

where the thermophoretic mobility is governed by particle dimensions, surface chemistry (e.g., charge density and hydrophobicity), and solvent properties.

**Figure 10:**
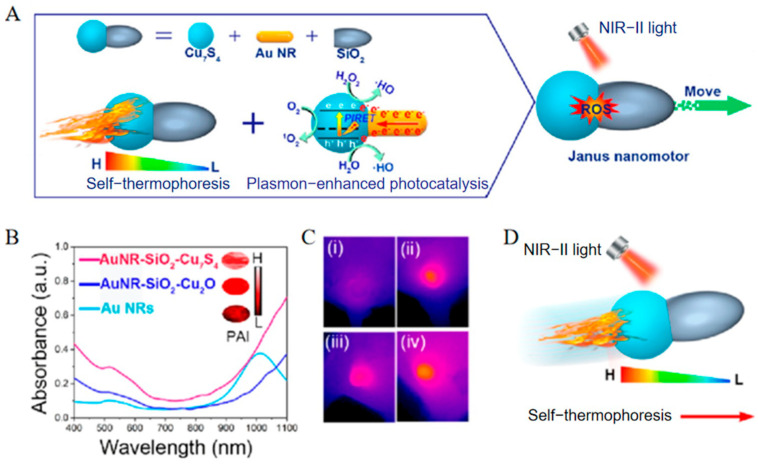
Schematic illustration of antimicrobial nanomotors: design and application. (**A**) Schematic illustration of the Janus architecture of AuNR-SiO_2_-Cu_7_S_4_ nanomotors and their plasmon-enhanced photocatalysis and self-thermophoresis behaviors after exposure to 1064 nm light. (**B**) UV–vis–NIR absorption spectra of dual-plasmonic nanomotors versus controls. (**C**) Thermal images of (i) PBS, (ii) AuNR-SiO_2_, (iii) AuNR-SiO_2_-Cu_2_O, and (iv) AuNR-SiO_2_-Cu. (**D**) Self-thermophoresis mechanism under NIR-II irradiation. Reproduced with permission from ref. [29]. Copyright 2023, American Chemical Society.

Liu et al. [29] demonstrated this principle using AuNR-SiO_2_-Cu_7_S_4_ nanomotors featuring a dual-plasmonic heterojunction (Figure 10A). Under 1064 nm NIR-II laser irradiation, gold nanorods (Au NRs) and copper sulfide (Cu_7_S_4_) exhibit strong electromagnetic field coupling via localized surface plasmon resonance. This synergy enhances light absorption by 1.9-fold relative to controls and achieves 55.3% photothermal conversion efficiency at the Au-Cu_7_S_4_ interface (Figure 10B,C). The SiO_2_ hemisphere remains comparatively cool, establishing an axial temperature gradient. Reduced fluid density near the high-temperature zone (Cu_7_S_4_) initiates thermo-osmotic flow toward the low-temperature region (SiO_2_), thereby driving particle motion opposite to the fluid direction (Figure 10D).

### 4.3. Ultrasound-Propelled Systems

Ultrasound waves can non-invasively penetrate centimeter-scale biological tissues, which represents the most notable advantage of ultrasound-driven systems. Compared to chemical propulsion, ultrasound-driven mechanisms avoid the biotoxicity associated with chemical fuels, thereby achieving superior biocompatibility. In contrast to light-driven approaches, which are limited by shallow penetration depth, ultrasound utilizes mechanical waves that overcome the physical constraints of optical transmission in biological tissues. This enables the delivery of therapeutic agents to regions that are otherwise difficult to access by other methods.

Asymmetric bubble recoil based on an ultrasound propulsion mechanism represents a pivotal technology for driving nanomotors. Its core principle relies on employing ultrasonic waves to trigger localized chemical reactions for gas generation, thereby achieving directional propulsion through precise control over bubble dynamics. Within this system, ultrasound not only provides mechanical agitation but also induces gas production via acoustic cavitation effects or direct excitation of sonosensitizer decomposition. The intrinsic spatial asymmetry of the Janus structure plays a critical role in this process, guiding bubble nucleation, growth, and collapse to generate non-equilibrium interfacial forces, thereby efficiently converting acoustic energy into directional mechanical motion.

A paradigm developed by Ye et al. [56] exemplifies this mechanism: the asymmetric Au NR-mSiO_2_/AIPH nanomotor features a spatially heterogeneous architecture comprising a gold nanorod (Au NR) partially enveloping a mesoporous silica (mSiO_2_) component. The mSiO_2_ hemisphere incorporates the sonosensitizer AIPH, while the Au NR side provides near-infrared-II (NIR-II) photoacoustic responsiveness. Under ultrasonic irradiation (1.0 MHz, 2.5 W/cm^2^), sonolysis of AIPH within the mesoporous silica simultaneously liberates nitrogen microbubbles and alkyl radicals. Nitrogen bubbles nucleate preferentially within the mesopores of the mSiO_2_ domain, detaching upon reaching critical size. When these bubbles collapse near the asymmetric material boundary, structural asymmetry at the mSiO_2_/Au interface induces microjetting directed toward the exposed Au NR side. The recoil momentum from these microjets ultimately propels the nanomotor’s directional locomotion.

Through ultrasound propulsion, these nanomotors can actively penetrate tumor tissue, overcoming the limitation of traditional nanomedicines that rely on passive diffusion, thereby achieving deep-tissue drug delivery.

### 4.4. Enzyme-Catalyzed Reactions

Enzyme catalysis is extensively studied in nanomotor systems and often constitutes a distinct category. Among the three aforementioned propulsion mechanisms, enzymatic catalysis, owing to its inherent nature as a natural protein, exhibits the most favorable biocompatibility. Since enzyme-driven systems can utilize endogenous substrates already present in vivo as fuel, they eliminate the need for externally supplied harmful chemicals. Furthermore, enzyme-powered nanomotors are capable of demonstrating chemotactic behavior in response to substrate concentration gradients, thereby indicating greater potential for clinical translation.

The core propulsion mechanism of enzyme-powered nanomotors lies in their asymmetric design, which converts biomolecular energy into mechanical motion. Within asymmetric micro/nanostructures, spatially selective immobilization of enzymes (e.g., urease and catalase) generates substrate-derived product concentration gradients or localized fluid perturbations, thereby inducing self-propulsion. This asymmetric driving force was first experimentally confirmed by Sen’s team through single-enzyme diffusion studies [58]. Subsequently, Sánchez’s group extended this principle to mesoporous silica carriers, demonstrating that random yet asymmetric enzyme distribution on particle surfaces breaks symmetry to enable autonomous motion [59]. Notably, current research predominantly focuses on aqueous environments, with limited exploration of enzyme motor mechanisms at oil/water interfaces or within oil phases. This gap in understanding motion mechanisms at these non-aqueous interfaces is now being addressed by the emerging paradigm of interface-catalyzed propulsion.

**Figure 11:**
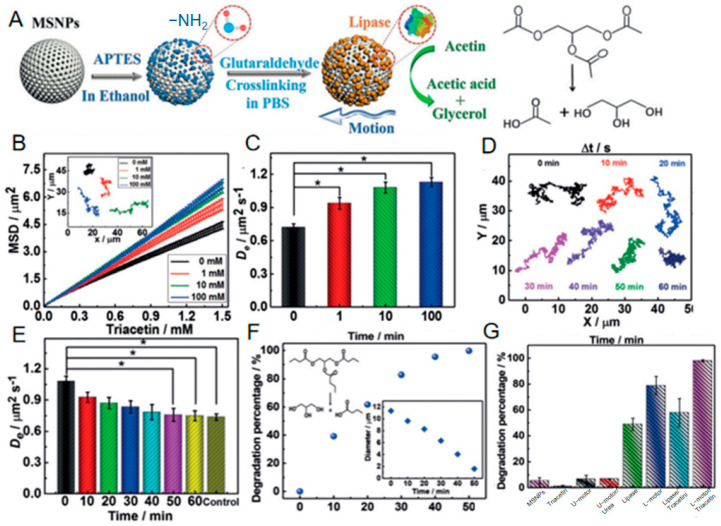
Design, motion characteristics, and degradation efficiency of lipase-powered mesoporous silica nanomotors (LNMs). (**A**) Schematic of lipase immobilization on MSNPs via glutaraldehyde crosslinking. (**B**) Representative trajectories of LNMs at varying triacetin concentrations. (**C**) Effective diffusion coefficients showing fuel concentration-dependent motion enhancement. (**D**) Sustained enhanced diffusion of LNMs over 40 min with 10 mM triacetin fuel. (**E**) Dynamic size reduction of a tributyrin droplet during LNM-mediated degradation. * *p* < 0.05 when compared to the control group. (**F**) Degradation efficiency comparison under different conditions after 1 h. (**G**) Showing the degradation percentage of tributyrin droplets under different conditions after 1 h. Reproduced with permission from ref. [30]. Copyright 2019, Wiley-VCH.

Wang et al. [30] immobilized lipase randomly onto mesoporous silica nanoparticles (MSNPs) via glutaraldehyde crosslinking (Figure 11A), leveraging molecular-scale asymmetry in enzyme distribution to generate asymmetric forces [60]. Using triglycerides (e.g., triacetin) as fuel, lipase-catalyzed hydrolysis created localized fluid disturbances and proton diffusion gradients, significantly enhancing particle Brownian motion. This manifested as an increased diffusion coefficient of 1.08 μm^2^/s with 50% higher than fuel-free systems (Figure 11C), demonstrating high-efficiency fluidic energy transduction. Crucially, this mechanism not only aligns with classical asymmetric enzyme motor kinetics (Patino et al.) but also exploits lipase’s inherent amphiphilicity for unique functional synergy: during propulsion, motors spontaneously anchor at oil/water interfaces of insoluble triglyceride droplets (e.g., tributyrin). Under confined diffusion at the interface (diffusion coefficient: 0.174 μm^2^/s), they continuously catalyze substrate hydrolysis. This tripartite asymmetric mechanism—integrating propulsion, interfacial localization, and degradation—achieved 98% droplet degradation within 50 min. This performance substantially surpasses free enzyme systems (57.9%) and other enzyme motors (e.g., urease motors < 7%), which have demonstrated high application value in addressing gastrointestinal mucus barriers that impede drug absorption by intestinal epithelial cells, validating the synergistic advantage of asymmetric propulsion and interfacial catalysis in complex multiphase systems (Figure 11G).

The aforementioned work has successfully demonstrated an efficient integrated synergy of “motion–localization–degradation” for enzymatic nanomotors operating at oil/water interfaces. This achievement establishes a robust paradigm for deploying asymmetric enzyme motors in complex environments, particularly those involving interfaces with insoluble substrates or products. However, most current propulsion strategies rely on introducing exogenous chemical fuels (e.g., urea and hydrogen peroxide), which inherently constrains their universality and sustainability in physiological settings, especially for biomedical applications.

In response to this limitation, researchers are actively exploring endogenous biomolecules as novel fuel sources for motor propulsion. These molecules are either inherently present or disease associated within biological systems.

**Figure 12:**
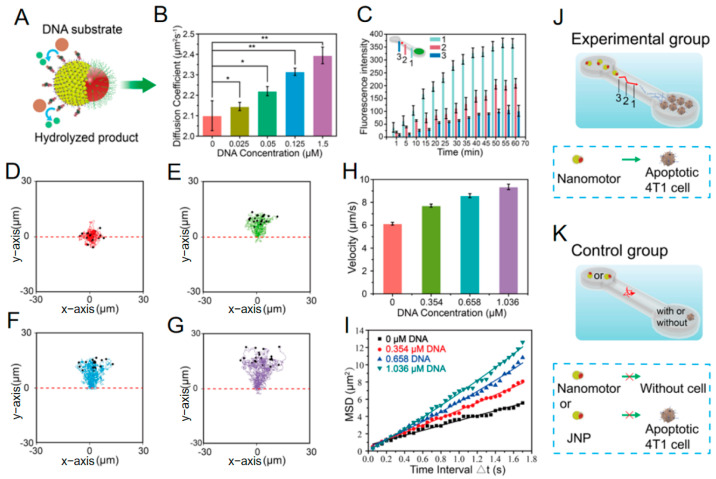
Motion dynamics of asymmetric nanomotors. (**A**) Schematic illustration of DNase functionalized nanomotor powered by DNA. (**B**) Diffusion coefficient of nanomotors with different concentrations of DNA as measured by dynamic light scattering (DLS), and data are shown as mean ± SD (*n* = 3) (Statistical analysis of significance value was presented: (* *p* < 0.05 and ** *p* < 0.01)). (**C**) Change in fluorescence intensity over time from position 1 to position 3, detected by the fluorescent probe PI. (**D**–**G**) Tracking paths of 100 consecutive frames of nanomotors in the presence of gel soaking in different concentrations of DNA. Twenty particles were analyzed in each group. (**D**) 0 μM, (**E**) 0.354 μM, (**F**) 0.658 μM, and (**G**) 1.036 μM. (**H**) The average velocity of nanomotors in different concentration of DNA corresponding to the tracking paths. (**I**) Average mean MSD analysis of nanomotors. (**J**) Schematic illustration of the experimental group for chemotaxis evaluation of nanomotors with 4T1 cells that pretreated with hyperthermia to induce apoptosis and DNA gradient in the chamber. (**K**) Schematic illustration of the control group with either nanomotor but no cells or of JNPs with apoptotic 4T1 cells in the chamber. Reproduced with permission from ref. [40]. Copyright 2021, American Chemical Society.

Demonstrating this paradigm shift, Ye et al. [40] developed an apoptotic tumor DNA-activated asymmetric nanomotor system exploiting DNase-catalyzed DNA hydrolysis to achieve coordinated enzyme-driven and thermophoretic propulsion. This design innovatively utilizes DNA as both fuel and chemoattractant: First, asymmetric functionalization of DNase on the polyacrylamide (PAA) hemisphere catalyzes DNA hydrolysis to generate nucleotide gradients, initiating diffusiophoretic motion. Concurrently, transient heat released during enzymatic reactions creates localized thermal fields, further accelerating motor migration via self-thermophoresis (Figure 12). This dual-mechanism synergy enables gradient sensing and autonomous navigation toward apoptotic tumor cell regions at ultra-low DNA concentrations (nM to μM range). Compared to prior enzyme motors dependent on high exogenous fuel concentrations [61], this work represents the first demonstration of (1) chemotactic motion powered by disease-associated endogenous DNA fuel and (2) validated self-navigating targeting capability within authentic tumor microenvironments, establishing a novel paradigm for tumor theranostics.

### 4.5. Multi-Phoretic Propulsion

While preceding sections focused on controlling individual propulsion mechanisms, complex biological environments demand nanomotors with multimodal synergy and adaptive switching capabilities. A key challenge in achieving such synergy lies in the precise regulation of directional conflicts and dynamic balance between different propulsion mechanisms.

**Figure 13:**
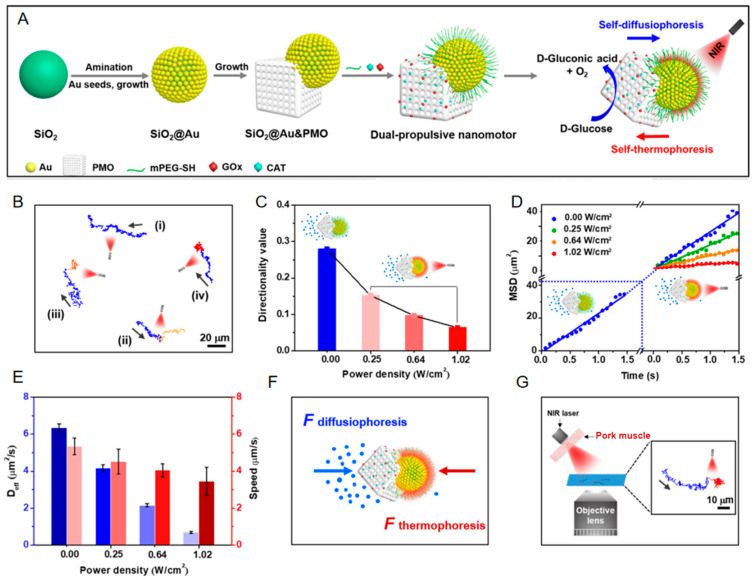
Schematic illustration of glucose-driven dual-propulsive Janus nanomotors: synthesis, NIR-regulated motion dynamics, and biological applicability. (**A**) Schematic illustration of the fabrication process and speed regulation mechanism of the dual-propulsive asymmetric nanomotor. (**B**) Time-lapse trajectories (in 24 s) of the typical glucose-driven nanomotors in 100 mM glucose solution before (blue line, in 12 s) and after (red line, in 12 s) the NIR irradiation with different power density: (i) 0.00, (ii) 0.25, (iii) 0.64, (iv) 1.02 W/cm^2^ (**C**) Directionality values, (**D**) MSD, and (**E**) the corresponding Deff and speeds of the glucose-driven nanomotors under the NIR irradiation with different power densities. (**F**) Scheme illustration of force analysis for the glucose-driven asymmetric nanomotor with NIR optical brake. (**G**) The in vitro experiment of the asymmetric nanomotor in biological tissue. Reproduced with permission from ref. [57]. Copyright 2022, American Chemical Society.

Addressing this challenge, Liu et al. [57] introduced an “NIR-optical braking” concept through precisely engineered mesoporous asymmetric nanomotors with bidirectional propulsion capabilities (Figure 13A). Within the periodic mesoporous organosilica (PMO) domain, glucose oxidase/catalase (GOx/CAT) enzymes are incorporated into the SiO_2_@Au&PMO–enzyme asymmetric nanocomposites (~350 nm diameter). This enzymatic system catalyzes glucose to generate asymmetric chemical gradients, propelling the nanomotors toward the Au nanoshell hemisphere. Conversely, near-infrared (NIR) light excitation of the SiO_2_@Au core–shell structure creates localized thermal gradients, driving thermophoretic motion toward the PMO domain. These counter-directional forces establish a dynamic antagonistic propulsion system (Figure 13F). This platform synergizes efficient enzymatic catalysis with optically regulated reverse thermophoresis. This combination enables remote precision control of nanomotor velocity under physiological glucose concentrations (Figure 13E), thereby overcoming fundamental limitations in controlling enzyme-powered motors within steady-state biological environments.

While the optical brake strategy pioneered by Liu et al. provides an elegant solution for remotely tuning propulsion speed via counter-directed forces, the reliance on external light sources inherently limits its applicability in deep tissues or scenarios requiring device-free operation. Indeed, approaches utilizing external stimuli like light or magnetism for dual-mode modulation [62] remain fundamentally constrained by limited tissue penetration depth and device dependency. Consequently, bio-endogenous stimuli (e.g., pH)-driven multimodal switching holds greater promise for clinical translation due to its inherent biocompatibility and self-contained operation within physiological contexts. However, prior demonstrations of pH-responsive nanomotors were primarily limited to regulating a single propulsion mode (e.g., bubble recoil or self-diffusiophoresis) [63,64], falling short of achieving the complex, adaptive multimodal synergy demanded by intricate biological environments.

**Figure 14:**
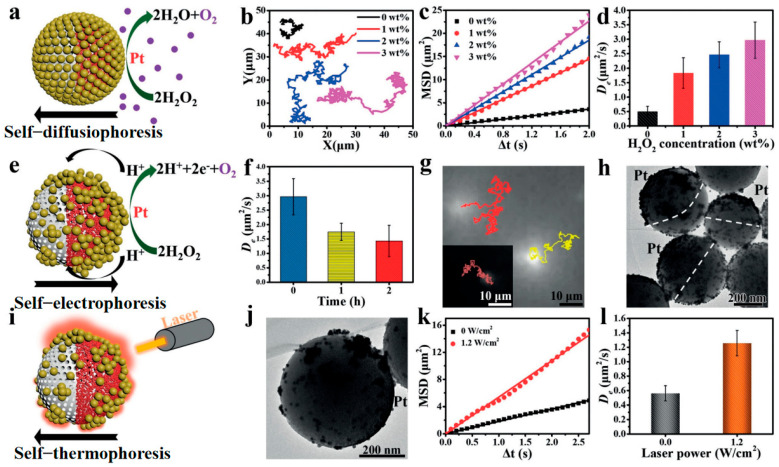
Schematic and experimental analysis of nanomotor propulsion: self-diffusiophoresis, switchable self-electrophoresis, and light-triggered self-thermophoresis dynamics with real-time degradation profiling. (**a**) Self-diffusiophoresis (H_2_O_2_ gradient); (**b**,**c**) trajectories and MSD; (**d**) De; (**e**) switched self-electrophoresis; (**f**) De in 3% H_2_O_2_ vs. time; (**g**) self-electrophoresis trajectories (2 h H_2_O_2_; inset: Brownian); red, nanomotors with larger optical spots; yellow, other nanomotors. (**h**) TEM after 2 h depletion; (**i**) light-induced self-thermophoresis; (**j**) TEM after 6 h H_2_O_2_; (**k**,**l**) MSD and De of light-driven motors (6 h laser + H_2_O_2_ depletion). Reproduced with permission from ref. [65]. Copyright 2020, Wiley-VCH.

To tackle this issue, Xing et al. [65] developed core@satellite asymmetric mesoporous silica-Pt@Au (JMPA) nanomotors, achieving the first pH-triggered tridirectional propulsion mode switching. This work represents an unprecedented advancement in dynamic asymmetry reconfiguration and multi-mechanism synergy. Leveraging pH-sensitive Cu^2+^ coordination bonds in weakly acidic H_2_O_2_ environments, the system induces dissociation of uniformly distributed gold nanoparticles (AuNPs) and their subsequent reorganization into Janus-type aggregates. This asymmetry evolution process sequentially activates three distinct propulsion mechanisms: Initially, Pt-AuNP spatial isolation triggers self-diffusiophoresis (Figure 14a–d). Upon AuNP reassembly near the Pt domain forming electrical pathways, propulsion switches to self-electrophoresis (Figure 14e–h). Ultimately, NIR laser irradiation generates asymmetric thermal gradients across the asymmetric AuNP aggregates, driving self-thermophoretic motion (Figure 14i–l). Finite element analysis confirmed enhanced thermophoretic force (0.81 pN vs. 0.11 pN), improving cancer cell membrane adhesion efficiency. This design enables dynamic asymmetry regulation via a single biological stimulus (pH), facilitating sequential activation of self-diffusiophoresis, self-electrophoresis, and self-thermophoresis. It establishes a new paradigm for intelligent nanomachines with multitask adaptability in biomedicine.

## 5. Asymmetric Nanomotors

Asymmetric nanomotors demonstrate transformative potential across multiple technological domains, fundamentally driven by structural asymmetry-enabled autonomous propulsion coupled with optimized energy conversion efficiency to achieve targeted functional outputs in biomedical theranostics, precision environmental remediation, and microscale electronics repair. This section systematically analyzes application mechanisms, technological evolution, and translational challenges of asymmetric nanomotors across three critical domains, starting with fundamental structural asymmetry characteristics and energy transduction principles. Through integration of core capabilities—namely autonomous propulsion, micro/nanoscale maneuverability, and tunable surface chemistry—this analysis establishes a foundational framework for interdisciplinary collaborative design.

### 5.1. Asymmetric Nanomotors in Biomedicine

Biomedicine represents the most revolutionary and rapidly advancing application domain for asymmetric nanomotors. The complex microenvironment of biological systems and the critical demand for precise intervention align synergistically with asymmetric nanomotors’ unique capabilities, including active targeting, controllable propulsion, drug delivery, and energy conversion. Nevertheless, clinical translation faces dual barriers: (1) long-term toxicity and immunogenicity triggered by synthetic components may disrupt physiological homeostasis, and (2) dependence on exogenous energy sources (e.g., light/magnetic fields) limits accessibility to deep-seated lesions. A pivotal strategy involves endogenous energy integration: by coupling with biofuel cells or leveraging lesion-specific microenvironmental molecules, asymmetric nanomotors can establish self-sustaining theranostic systems. For instance, recent studies confirm that urate oxidase-functionalized asymmetric nanomotors synchronously convert uric acid in gout foci into hydrogen peroxide, achieving simultaneous self-propulsion and metabolic clearance [66].

Consequently, this section focuses on the frontier biomedical applications and challenges of asymmetric nanomotors. These span diverse tumor treatment strategies, disease-specific therapeutics, and minimally invasive surgical assistance.

**Figure 15:**
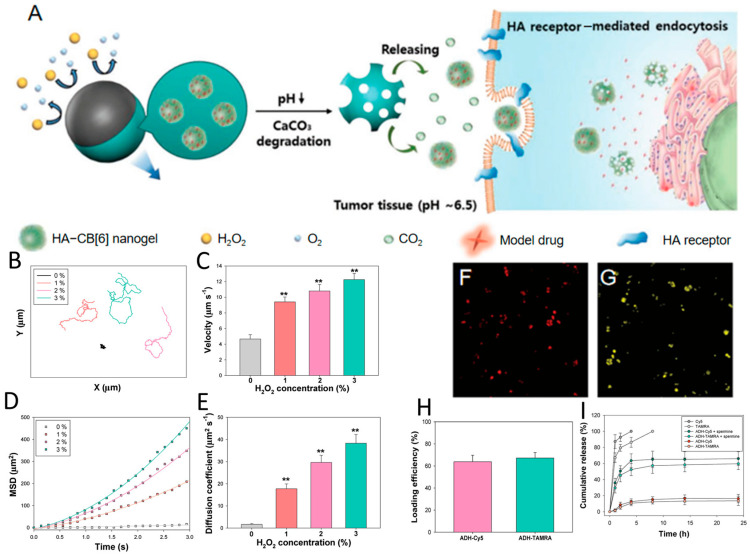
Nanomotors for intracellular drug delivery: dual-activation mechanism, fuel-concentration-dependent propulsion dynamics, and validated drug loading/release performance. (**A**) Schematic illustration for the intracellular delivery of model drugs loaded in the nanomotors by the dual stimuli. (**B**) The trajectories of representative nanomotors for 30 s in the presence of hydrogen peroxide. (**C**) The velocity with increasing concentration of hydrogen peroxide. (**D**) The mean square displacement with increasing time interval, analyzed from x- and y-coordinate tracking of 20 particles at each condition. (**E**) The diffusion coefficient values calculated from the MSD. Confocal microscopic images of Pt/CaCO_3_@HA-CB nanomotors loaded with (**F**) ADH-Cy5 and (**G**) ADH-TAMRA (** *p* ≤ 0.01, with H_2_O_2_ vs without H_2_O_2_). (**H**) The loading efficiency of model drugs determined by UV–vis–NIR spectroscopy. (**I**) In vitro release tests of model drugs for 24 h at the following conditions. Reproduced with permission from ref. [41]. Copyright 2019, Wiley-VCH.

For tumor therapy, Choi et al. [41] developed a platinum-catalyzed (Pt) degradable nanomotor based on a calcium carbonate core and cucurbit (CB) uril-conjugated hyaluronate (Pt/CaCO_3_@HA-CB) for targeted drug delivery to tumors. Its antitumor efficacy relies on a dual-stimuli-responsive mechanism: (1) Within the acidic tumor microenvironment (pH 6.5), the CaCO_3_ core dissolves to release HA-CB nanogels (~300 nm) (Figure 15A). (2) Liberated HA undergoes receptor-mediated endocytosis for efficient tumor cell uptake (e.g., in MDA-MB-231 cells (MD Anderson-Metastatic Breast-231)). The Pt coating exploits tumor-overexpressed H_2_O_2_ for oxygen-generation-driven propulsion, enhancing drug delivery efficiency (Figure 15B–E). Model drugs were modularly loaded via CB-polyamine host–guest interactions, achieving >60% encapsulation efficiency and exhibiting controlled release in the presence of spermine as a glutathione (GSH) competitor (Figure 15F–I). Cellular assays confirmed nanomotor degradation within 2 h and effective delivery of both hydrophilic/hydrophobic agents to cancer cells, a capability unattainable by traditional SiO_2_/Pt micromotors due to non-internalizable dimensions. This system integrates tumor microenvironment-responsive propulsion, active targeting, and spatiotemporally controlled drug release, establishing a novel strategy for intelligent anticancer drug delivery.

Integrated cancer theranostics: this study engineered a glutathione (GSH)-chemotactic nanomotor (designated PMG NMs) based on an aminated metal–organic framework (NH_2_-MIL-101), achieving tumor targeting and ferroptosis therapy through asymmetric modification with methoxy polyethylene glycol (mPEG) and γ-glutamyl transpeptidase (GGT). The antitumor mechanism involves triple synergistic actions: (1) Chemotactic Navigation and Deep Penetration: GGT catalyzes GSH hydrolysis to establish a directional diffusion gradient, while the mPEG tail stabilizes propulsion trajectory. This enables nanomotor migration along the GSH concentration gradient toward tumors, yielding 9.6-fold higher tumor accumulation than passively diffusing nanoparticles. The system achieves 70 μm penetration depth in multicellular tumor spheroids (MTSs). (2) Synergistic GSH Depletion: Fe^3+^ nodes in NH_2_-MIL-101 are reduced to Fe^2+^ by GSH, consuming intracellular thiol reserves. Concurrent GGT-catalyzed GSH hydrolysis and ferroptosis inducer Erastin (loaded as PMG@E NMs) collectively deplete tumor GSH to 10.1% of baseline levels. (3) Ferroptosis Activation: Fe^2+^ ions drive Fenton reactions generating reactive oxygen species (ROS), elevating lipid peroxidation (malondialdehyde [MDA] levels increased 2.4-fold versus controls). This oxidative cascade triggers mitochondrial network fragmentation.

In vivo results demonstrate that PMG@E NMs prolong Erastin circulation time, nearly eliminating tumors within 24 days without systemic toxicity. This work pioneers the use of GGT-GSH biochemical reactions to construct chemotactic nanomotors, establishing a novel paradigm for tumor-specific theranostics [67].

Asymmetric nanomotors demonstrate unique advantages in treating specific diseases such as acute kidney injury (AKI), where conventional therapies face substantial limitations. Hydrophobic or metabolically unstable drugs exhibit poor bioavailability, while kidney-targeting agents remain scarce. Small-molecule therapeutics undergo rapid systemic clearance, failing to achieve therapeutic concentrations in injured renal tissue without high-dose administration that risks adverse effects. Consequently, developing novel delivery systems capable of efficient renal targeting, sustained therapeutic drug levels, and minimized toxicity is imperative. Tong et al. [24] recently addressed this challenge through an innovative carbon monoxide (CO)-propelled asymmetric nanomotor system designated Au@MSN/CORM-401@HA (AMCH).

**Figure 16:**
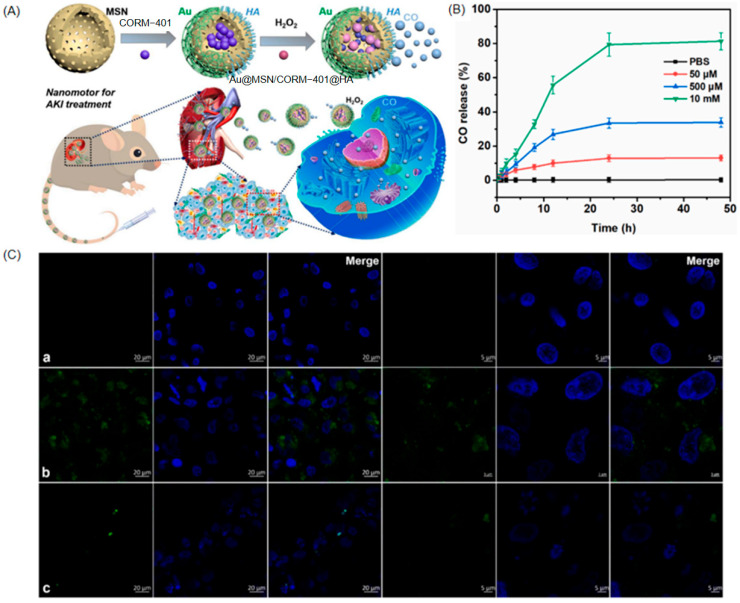
Schematic illustration and in vitro validation of Au@MSN/CORM-401@HA nanomotors for targeted CO delivery in acute kidney injury therapy. (**A**) Schematic illustration of Au@MSN/CORM-401@HA (AMCH) for the active delivery of CO to relieve AKI. (**B**) Characteristics of AMCH in vitro. (**C**) ROS measurement in vitro (a, normal HKC cells; b, AKI model cells; c, AMCH + AKI model cells) (DCFH-DA, DAPI and Merge). Reproduced with permission from ref. [24]. Copyright 2023, Elsevier.

The AMCH system employs an aminated mesoporous silica nanoparticle core loaded with a CO-releasing molecule (CORM-401) at 8.1 wt% loading capacity. Its surface features asymmetric functionalization with gold nanoparticles on one hemisphere and renal tubule-targeting hyaluronic acid on the opposite side. The therapeutic mechanism capitalizes on the pathologically elevated hydrogen peroxide microenvironment within AKI lesions. Initially, HA guides active targeting to injured renal tubular epithelium via CD44 receptor recognition (Figure 16A). Subsequently, local H_2_O_2_ triggers rapid CO release from CORM-401, achieving 84.6% release over 48 h under 10 mM H_2_O_2_ conditions (Figure 16B). Furthermore, asymmetric CO generation produces thrust that drives autonomous propulsion at 4.24 μm/s in 10 mM H_2_O_2_, increasing the diffusion coefficient fivefold compared to static conditions. This motion significantly enhances nanomotor–cell interactions and drug delivery efficiency.

In vitro studies using human renal tubular epithelial cells (HKCs) confirmed excellent biocompatibility. Therapeutically, AMCH substantially suppressed AKI-induced apoptosis, as confirmed by TUNEL staining. It concurrently mitigated oxidative stress damage by reducing reactive oxygen species levels, restoring mitochondrial membrane potential, and enhanced Na^+^/K^+^-ATPase activity (Figure 16C). In murine AKI models, intravenous administration (10 mg/kg) improved renal function, evidenced by significant reductions in blood urea nitrogen, serum creatinine, proteinuria, and renal injury biomarkers including neutrophil gelatinase-associated lipocalin and N-acetyl-β-D-glucosaminidase.

Taken together, a fundamental trade-off exists between propulsion efficiency and biocompatibility within biomedical applications. This conflict necessitates targeted compromises based on the specific characteristics of the pathological microenvironment. For instance, the sonosensitizer-powered nanomotor designed by Ye et al. achieved ±35 μm targeting precision in subcutaneous tumor models; however, its penetration depth was constrained by ultrasonic attenuation (≤5 mm), and the gold nanoparticle components posed risks of long-term retention [56]. This illustrates that superficial tissues may tolerate moderate risks associated with light-driven systems to achieve sub-millimeter manipulation precision. Conversely, deep-seated tumors require enzyme-catalytic mechanisms to ensure long-term safety, as exemplified by Wang’s lipase nanomotor, which utilized endogenous triglyceride fuel to achieve 98% degradation efficiency at oil/water interfaces without exogenous toxicity [30].

This requirement for spatiotemporal adaptation based on lesion location and properties further solidifies the central role of enzyme-catalytic propulsion in scenarios demanding deep-tissue penetration and long-term biocompatibility. Moreover, enzyme-catalytic drive leverages direct utilization of biomolecular energy (e.g., substrate catalytic gradients), granting it significant advantages in biocompatibility and microenvironmental adaptability, thereby establishing it as the preferred mechanism for in vivo applications. In contrast, light/chemical propulsion faces substantial in vivo limitations: light-driven systems depend on external equipment with poor tissue penetration, while chemical drives often require highly toxic fuels (e.g., H_2_O_2_). Consequently, multimodal cooperative propulsion emerges as an advanced strategy to overcome the limitations of singular mechanisms. For example, Xing’s pH-responsive JMPA nanomotor, through dynamic structural reconfiguration, sequentially activated self-diffusiophoresis, self-electrophoresis, and self-thermophoresis, significantly enhancing cancer cell adhesion efficiency [65].

In summary, the core of navigating these trade-offs and optimizing mechanisms lies in the precise regulation of the relationship between the structural asymmetry of the nanoparticles, the related energy conversion capability and corresponding functional application. Structural asymmetry (e.g., Janus heterojunctions and asymmetric enzyme distribution) forms the foundation for energy conversion, dictating the mode of driving force generation. Energy conversion efficiency must concurrently address biocompatibility constraints (e.g., utilization of endogenous fuels and low-intensity stimuli). Ultimately, the functional application (e.g., targeting efficiency and degradation rate) depends on the synergy between these two elements. This structure–activity relationship elevates the application-specific decision making into a universal design paradigm, guiding the development of intelligent nanosystems capable of operating within complex biological environments.

### 5.2. Environmental Remediation

Environmental pollution has emerged as one of the most urgent global public health challenges amid accelerated industrialization and the continuous emergence of novel contaminants. Conventional environmental remediation faces limitations including low diffusion efficiency and poor adaptability to complex media. Asymmetric nanomotors with autonomous propulsion offer a novel solution for efficient contaminant degradation and separation through enhanced mass transfer, emerging as a highly promising environmental remediation strategy. Their self-propulsion enables improved mixing, targeted delivery, and effective interactions with contaminants within complex matrices such as soil and wastewater. Crucially, the deliberate design of asymmetric morphologies confers directional motion and efficient propulsion mechanisms. This design, which includes structures such as tubes or complex concave shapes, also enables functionality in high-ionic-strength environments where traditional phoretic mechanisms fail. Optimizing this asymmetry is key to enhancing mobility, payload capacity, catalytic efficiency, and ultimately decontamination performance in real-world challenging scenarios. Recent research advances in nanomotor synthesis focus on precise particle structure control to overcome fundamental size limitations in propulsion or minimize hydrodynamic resistance, paving the way for more effective nanoscale environmental technologies.

A major challenge in nanomotor development has been downsizing bubble-propelled systems below 1 micron, a scale where excessive energy barriers inhibit bubble nucleation. To address this, Guan et al. developed a strategy based on controlled engineering of the platinum shell’s microstructure. They designed eccentric polystyrene–polydopamine@platinum (PS–PDA@Pt) Janus motors (PEMNMs) leveraging lattice mismatch effects between Pt and PDA. This approach generated densely distributed island-like platinum nanoparticles and nano-pits across the surface. These structural features significantly reduced bubble nucleation energy barriers and stabilized the gas–liquid–solid triple contact line, enabling efficient bubble propulsion even at reduced dimensions of 200 nm with speeds reaching 56.3 μm/s. Moreover, these PEMNMs exhibit robust propulsion in high-ionic-strength media (e.g., 150 mM NaCl), which is critical for remediation applications in saline wastewater or biosamples. This breakthrough not only validates the critical role of microstructure engineering in nanoscale bubble nucleation but successfully extends bubble propulsion mechanisms to the submicron scale, establishing a new paradigm for developing intelligent remediation systems applicable to miniaturized pollution scenarios such as soil micropore contaminant removal or in situ cellular toxin degradation.

**Figure 17:**
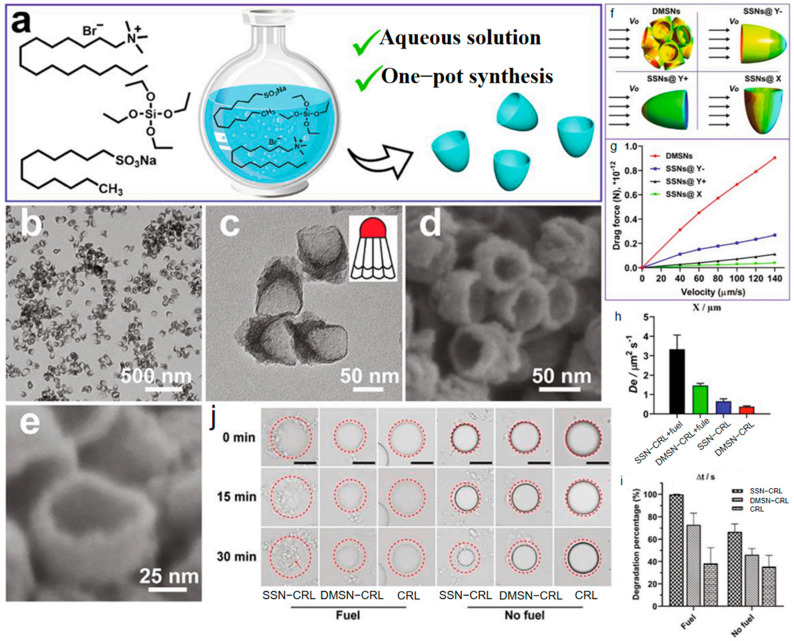
Asymmetric architecture, propulsion dynamics, and validated lipidolytic efficacy. (**a**) Schematic illustration of the one-pot self-assembly approach for large-scale synthesis of SSNs. (**b**) Low- and (**c**) high-magnification TEM images and (**d**,**e**) SEM images of SSNs. (**f**) Different flow directions with respect to various orientations of SSNs and DMSNs. (**g**) The relationship between drag force and velocity in different orientations of SSNs and DMSNs calculated by computational modeling using FEM. Single-nanoparticle tracking results. (**h**) Calculated diffusion coefficients from MSD curves. (**i**) Tributyrin degradation after 30 min. (**j**) Corresponding optical microscopy images showing near-complete droplet disappearance for SSN-CRL. Reproduced with permission from ref. [68]. Copyright 2021, Elsevier.

In contrast, Fu et al. [68] adopted a strategy centered on minimizing hydrodynamic drag to enhance nanomotor diffusivity and catalytic contact frequency with pollutants. They achieved gram-scale synthesis of shuttlecock-like silica nanoparticles (SSNs) via a facile one-step aqueous self-assembly process using a dual-surfactant system (CTAB/SDS) (Figure 17a). The unique asymmetric architecture—featuring a tapered streamlined body and a large open cavity (~65 nm) (Figure 17b–e)—significantly reduces drag forces during propulsion, as confirmed by finite element modeling (Figure 17f,g). Lipase (CRL) was efficiently encapsulated within the cavity to fabricate SSN-CRL nanomotors. Driven by substrate gradients (triacetin), these nanomotors exhibited approximately 2.2-fold higher diffusivity than their spherical dendritic mesoporous silica nanoparticle (DMSN) counterparts (Figure 17h–j). This enhanced mobility directly translated to superior catalytic performance. In tributyrin droplet degradation assays, SSN-CRL achieved near-complete degradation (100%) within 30 min when fuel was supplied, significantly outperforming DMSN-CRL (73%). This improvement underscores the critical role of nanostructural design in minimizing energy dissipation during motion, thereby maximizing the efficiency of enzyme-loaded nanomotors for pollutant decomposition in aquatic environments.

### 5.3. Asymmetric Nanomotors in Microelectronics Repair and Soldering

The ongoing miniaturization and heightened integration of microelectronic devices present formidable challenges for circuit repair at microscopic scales. Conventional soldering techniques are constrained by thermal diffusion effects and limited spatial resolution, impeding precise restoration of nanostructures, while static fillers lack autonomous defect localization capabilities in complex networks. Asymmetric nanomotors have recently emerged as a transformative paradigm for microelectronics repair, leveraging their controllable self-propulsion and environmental responsiveness. These motors harness catalytic reactions to generate self-electrophoretic or bubble-propulsive forces, enabling autonomous navigation to target sites at microscopic scales, potentially realizing next-generation intelligent soldering materials. Nevertheless, existing systems remain predominantly limited to rigid materials with inherent constraints in conductivity, deformation adaptability, and low-temperature soldering capability.

**Figure 18:**
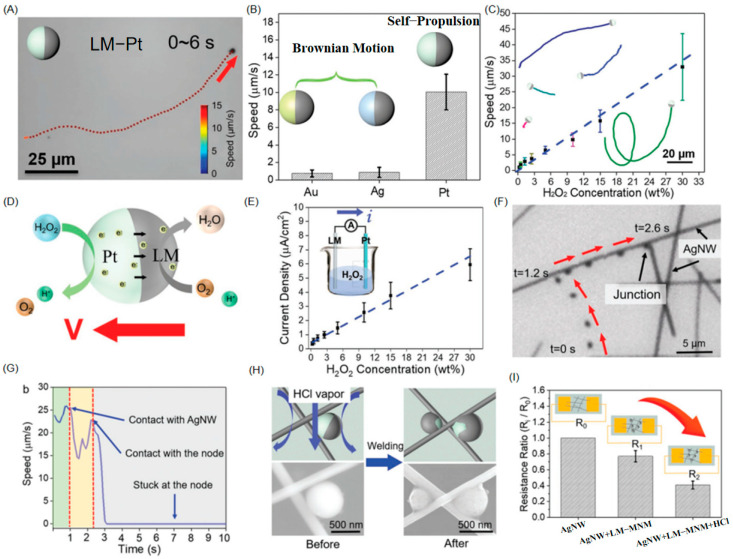
Motion mechanisms of nanomotors and validation of microwelding applications. (**A**) Platinum-coated liquid metal-based micro/nanomotor (Pt-coated LM-MNM) locomotion trajectories. (**B**) Velocity comparison for Au-, Ag-, and Pt-coated LM-MNMs in 10 wt% H_2_O_2_. (**C**) Pt-LM-MNM speed dependence on H_2_O_2._ concentration. (**D**) Self-electrophoretic propulsion mechanism: H_2_O_2._ oxidation (Pt side) and reduction (LM side). (**E**) Measured current density for LM–Pt system versus H_2_O_2._ concentration. (**F**) Targeted navigation of LM-MNM along AgNWs to junction. (**G**) Speed reduction during junction localization. (**H**) HCl-vapor-enabled junction microwelding (schematic and SEM validation). (**I**) Reduced normalized resistance (R/R_0_) after LM-MNM addition (R_1_) and welding (R_2_). Reproduced with permission from ref. [43]. Copyright 2019, Wiley-VCH.

Addressing these limitations, Wang et al. [43] achieved a significant advance through liquid metal-based asymmetric nanomotors. This pioneering work integrates liquid metal (Galinstan alloy) with self-propulsion technology to create nanomotors combining autonomous targeting and intelligent welding functionalities. They utilized an ultrasonication–sputtering technique to fabricate Pt-based asymmetric liquid metal motors (down to submicron dimensions). This process constructed a semi-encapsulated Pt heterostructure via interfacial engineering between liquid metal (Ga-In-Sn alloy) and platinum. The spontaneously formed 2-nm Ga_2_O_3_ surface layer exhibits dual electron-tunneling conductivity and acid-responsive solubility. Propelled by proton gradients from Pt-catalyzed H_2_O_2_ decomposition, these motors autonomously migrate along silver nanowires (AgNWs) (Figure 18A–E) to junction points (Figure 18F,G). Subsequent acid vapor exposure (pH 0.4) dissolves the Ga_2_O_3_ layer, releasing liquid metal to form metallic bonds at junctions, enabling in situ intelligent welding (Figure 18H,I). This design pioneers the operation of chemically propelled liquid metal motors at submicron scales (<1 μm), resolving persistent challenges in junction resistance within micro/nanocircuits while outperforming conventional rigid motors through superior conductivity and room-temperature soldering capability. It establishes a scalable platform for nanoscale in situ repair of microelectronic devices.

## 6. Summary and Future Perspectives

Asymmetric nanomotors have evolved from fundamental motion prototypes to functionally integrated systems, demonstrating transformative potential across biomedical, environmental, and microelectronic domains. In synthesis, template-assisted strategies (soft/hard templates) enable precise structural control of bowl-shaped mesoporous silica and hollow carbon spheres, while physicochemical approaches achieve single-step asymmetric fabrication through thermodynamic regulation of interfacial energy. Various advanced techniques have been developed for the fabrication of asymmetric nanoparticles, including Pickering emulsion, confined synthesis and vapor deposition, etc.

Research on propulsion mechanisms has transcended traditional chemical-driven frameworks. This progress is evidenced by innovative systems such as self-thermophoresis in plasmonic heterojunctions, enzyme-catalyzed chemotactic behavior, and stimulus-responsive propulsion mode switching. Notable advances include pH-triggered tridirectional propulsion mode and near-infrared optical braking systems capable of real-time velocity modulation under physiological conditions.

Therapeutic applications leverage autonomous navigation for enhanced targeting: glutathione-chemotactic nanomotors achieve tumor accumulation exceeding 25% ID/g, while carbon monoxide-propelled systems reinforce renal tubule repair through hydrogen peroxide-triggered autonomous motion. Environmental remediation benefits from engineered asymmetric designs: submicron bubble-propelled motors overcome nucleation barriers in high-ionic-strength media, and shuttlecock-like architectures minimize hydrodynamic resistance to triple catalytic degradation kinetics.

In microelectronics, liquid metal-based asymmetric motors accomplish autonomous circuit localization and room-temperature soldering at submicron scales, providing an elegant solution to the persistent challenge of nanoscale junction resistance.

Notwithstanding these advancements, the clinical translation of asymmetric nanomotors remains impeded by several critical challenges: the suboptimal biocompatibility of metallic catalysts, inadequate navigation stability under dynamic physiological conditions, and insufficient understanding of their long-term toxicity and clearance mechanisms. Next-generation designs should prioritize the use of fully biodegradable materials, such as enzyme–polymer hybrids, and biohybrid systems incorporating cellular membranes to enhance stealth properties.

Future breakthroughs in asymmetric nanomotors will emerge from interdisciplinary integration aimed at addressing fundamental translational challenges. The incorporation of artificial intelligence (AI) has become essential for predictive design, leveraging machine learning algorithms to perform multiscale modeling of enzyme–substrate docking kinetics and nanoscale fluid interactions, thereby optimizing structure–propulsion–function relationships. Instead of relying solely on direct material design, researchers can now input desired functional specifications into AI systems. These models explore vast chemical and material spaces to generate numerous novel nanomotor configurations that meet predefined criteria, including unprecedented geometries, composite material combinations, surface functionalization strategies, as well as designs inspired by existing asymmetric nanomotors with analogous functions.

Furthermore, AI-driven automated synthesis platforms enable the standardized and reproducible fabrication of nanomotors. During production, machine learning algorithms leverage real-time data from dynamic light scattering (DLS), microscopy, and spectroscopy to tightly regulate manufacturing processes, ensuring strict compliance with quality specifications while supporting high-throughput automated manufacturing. In practical applications, AI algorithms further integrate real-time multimodal imaging data (e.g., MRI, fluorescence imaging, and ultrasound) to construct dynamic in vivo navigation systems. These systems not only track the spatial distribution and directional movement of nanomotor swarms but also monitor their velocity and even estimate local drug release profiles, thereby establishing a closed-loop technological framework from intelligent synthesis to precision application. This AI-driven nanomotor platform is driving the transition toward an era characterized by autonomous, adaptive, and transformative solutions across medical, environmental, and related fields.

## Figures and Tables

**Table 1:** Comparative analysis of synthesis method.

Synthesis Method	Throughput	Clinical Compatibility	Key Advantages/Limitations	Optimal Application Scenario	Reference
Soft-templating method	Low–medium	Moderate compatibility	Mild conditions; complex template removal; low yield	Targeted thrombolysis with biofuel	[44]
Hard-templating method	Medium	Limited compatibility	Precise morphology; hazardous HF etching; multi-step	Photothermal-chemotactic dual-propulsion drug delivery	[45]
Self-assembly synthesis approach	Low	Elevated compatibility	Dynamic structure adaptation; built-in stimuli responsiveness; complex osmotic/thermal control required	Light-responsive smart vesicles	[46]
Physical vapor deposition	Very low	Limited compatibility	Nanoscale precision; expensive equipment; low throughput	High-precision in vitro diagnostics	[47]
Pickering emulsion	High	Moderate compatibility	High drug loading; scalable production; ideal for drug delivery	Tumor-targeted drug delivery	[48]
Single-step, thermodynamic-controlled coating method	High	Moderate compatibility	Template-free; single step; low toxicity	Deep-tissue tumor penetration	[49]
Multi-step bioconjugation	Low	Exceptional compatibility	Optimal biocompatibility; complex multi-step process	Thrombus-targeted therapy	[50]

**Table 2:** Comparative analysis of propulsion mechanisms.

Propulsion Mechanism	Speed (μm/s)	Directional Control	Biocompatibility	Fuel/Energy Requirement	Reference
Chemical (H_2_O_2_)	3.85–7.15	High (0.76)	Poor	Exogenous H_2_O_2_	[55]
Light driven (NIR)	9.8	High	Good	NIR laser	[29]
Ultrasound propulsion	0.73–8.6	Medium (inherent directionality)	Good	Embedded chemical reactant	[56]
Enzyme catalyzed	6.13–9.35	Medium (chemotactic behavior)	Excellent	Endogenous fuels	[40]
Multimodal	3.46–6.49	Precision	Good	Hybrid input	[57]
